# Small Molecule Cjoc42 Improves Chemo-Sensitivity and Increases Levels of Tumor Suppressor Proteins in Hepatoblastoma Cells and in Mice by Inhibiting Oncogene Gankyrin

**DOI:** 10.3389/fphar.2021.580722

**Published:** 2021-03-04

**Authors:** Amber M. D’Souza, Ashley Cast, Meenasri Kumbaji, Maria Rivas, Ruhi Gulati, Michael Johnston, David Smithrud, James Geller, Nikolai Timchenko

**Affiliations:** ^1^ Departments of Oncology, Cincinnati Children’s Hospital Medical Center, Cincinnati, OH, United States; ^2^Department of Pediatrics, Hematology and Oncology, University of Illinois College of Medicine, Peoria, IL, United States; ^3^ Departments of Surgery, Cincinnati Children’s Hospital Medical Center, Cincinnati, OH, United States; ^4^ Department of Surgery, University of Cincinnati, Cincinnati, OH, United States; ^5^ Department of Chemistry, University of Cincinnati, Cincinnati, OH, United States

**Keywords:** hepatoblastoma (HBL), cjoc42, tumor suppressors, oncogene, liver cancer

## Abstract

**Objective:** Relapsed hepatoblastoma (HBL) and upfront hepatocellular carcinoma (HCC) are notoriously chemoresistant tumors associated with poor outcomes. Gankyrin (Gank) is a known oncogene that is overexpressed in pediatric liver cancer and implicated in chemo-resistance. The goal of this study was to evaluate if the Gank-tumor suppressor axis is activated in chemoresistant hepatoblastoma patients and examine if an inhibitor of Gank, Cjoc42, might improve the chemosensitivity of cancer cells.

**Methods:** Expression of Gank and its downstream targets were examined in fresh human HBL samples using immunostaining, QRT-PCR, and Western Blot. Cancer cells, Huh6 (human HBL) and Hepa1c1c7 (mouse HCC) were treated with Cjoc42 and with Cjoc42 in combination with cisplatin or doxorubicin. Cell proliferation, apoptosis, and chemoresistance were examined. To examine activities of Cjoc42 *in vivo*, mice were treated with different doses of Cjoc42, and biological activities of Gank and cytotoxicity of Cjoc42 were tested.

**Results:** Elevation of Gank and Gank-mediated elimination of TSPs are observed in patients with minimal necrosis after chemotherapy and relapsed disease. The treatment of Huh6 and Hepa1c1c7 with Cjoc42 was not cytotoxic; however, in combination with cisplatin or doxorubicin, Cjoc42 caused a significant increase in cytotoxicity compared to chemotherapy alone with increased apoptosis. Examination of Cjoc42 in WT mice showed that Cjoc42 is well tolerated without systemic toxicity, and levels of tumor suppressors CUGBP1, Rb, p53, C/EBPα, and HNF4α are increased by blocking their Gank-dependent degradation.

**Conclusions:** Our work shows that Cjoc42 might be a promising adjunct to chemotherapy for the treatment of severe pediatric liver cancer and presents mechanisms by which Cjoc42 increases chemo-sensitivity.

## Introduction

Hepatoblastoma (HBL) is the most common primary pediatric hepatic malignancy (Aronson and Meyer, 2016). Treatment strategies have evolved significantly over the last 25 years with better outcomes achieved by cisplatin-based chemotherapy regimens, however, 30% of patients will eventually relapse or succumb to the disease due to chemoresistance (Hiyama, 2014; Czauderna and Garbier, 2018). The use of targeted therapies based on tumor biology may improve treatment and survival. Preliminary work identified Gankyrin (Gank) as one of the key molecules elevated in HBL leading to tumor growth by triggering degradation of tumor suppressor proteins (TSPs) Rb, p53, C/EBPα, HNF4α, and CUGBP1 (Lewis et al., 2017; Cast et al., 2018; D’Souza et al., 2018; Valanejad et al., 2018). Intensive investigations of Gank have also identified multiple pathways by which this oncogene might promote liver cancer (Timchenko and Lewis, 2015). These pathways include the elimination of tumor suppressors (Wang et al., 2010; D’Souza et al., 2018; Valanejad et al., 2018; Higashitsuji et al., 2000; Dawson, 2008) activation of beta-catenin/WNT signaling (He et al., 2016; Liu et al., 2019), and activation of stem-cell markers and several key liver cancer-related genes such as OCT4 and c-myc (Qian et al., 2012; Valanejad et al., 2017; Liu et al., 2019). Several investigators recently showed that Gank activates liver proliferation in animal models and patients with non-alcoholic fatty liver disease (NAFLD) (Huang et al., 2017; Sakurai et al., 2017). Gank also degrades TSPs in different liver diseases leading to increased liver proliferation and development of liver cancer, NAFLD, and fibrosis (Lewis et al., 2017; Nguyen et al., 2018; Cast et al., 2019).

Gank has been shown to be linked to chemoresistance in several different types of malignancies (Song et al., 2013; Sakurai et al., 2017; Zamani et al., 2017; Li et al., 2018). Additionally, inhibition of Gank has been shown to reduce cell proliferation and has been suggested as a potential target for tumor therapy (Song et al., 2013; Zamani et al., 2017; Li et al., 2018; Sun et al., 2018). Cjoc42 is a small molecule binder of Gank (Chattopadhyay et al., 2016). This compound inhibits the ability of Gank to interact with other proteins, including TSPs, and leads to reduced liver cancer cell proliferation *in vitro* (D’Souza et al., 2018). Since many aggressive cases of HBL are characterized by strong activation of Gank-TSP pathways, presented herein, we undertook a further investigation in the pharmacologic modulation of these pathways. We demonstrate that many cases of hepatoblastoma have a strong activation of the Gank-TSPs pathway and that Cjoc42 enhances the cytotoxicity of traditional chemotherapeutic agents used in pediatric liver cancer. Cjoc42 appears to be safe and tolerable in mice in doses that are sufficient to protect TSPs from Gank-mediated degradation. Our data suggests that Cjoc42 might be a promising adjunctive treatment for children with chemoresistant liver cancer.

## Materials and Methods

### Animals

Experiments with animals were approved by the Institutional Animal Care and Use Committee at Cincinnati Children’s Hospital (protocol IACUC2014-0042). Wild type (C57BL) mice were utilized for Cjoc42 toxicity studies.

### Immunohistochemistry

Liver sections were fixed overnight in 4% PFA, embedded in paraffin, and sectioned (6 µm sections). IHC for Gank (Sugnam WH0005716M1) was completed on 10 hepatoblastoma samples. The sections were scored independently by three observers based on both the proportion of positively stained tumor cells and the intensity of staining. Three fields were examined per patient at 10x magnification. The proportion of positive tumor cells was scored as follows: 0, no positive tumor cells; 1, < 10%; 2, 10%–35%; 3, 35%–75%; 4, > 75%. The intensity of staining was graded according to the following criteria: 1, no staining; 2, weak staining (light yellow); 3, moderate staining (yellow-brown); 4, strong staining (brown). The degree of Gank immunostaining was defined as the proportion score multiplied by the staining intensity score, with SI ≥ 8 was defined as high Gank expression, and SI < 8 was defined as low Gank expression.

### Clinical and histological data on pediatric hepatoblastoma patients

After IRB approval, clinical and histological data were collected by a clinician (AMD) using the electronic medical record system. Histology and % necrosis were obtained from pathology reports. Chemoresistance was defined as < or = 50% necrosis at the time of resection. Relapse was defined as recurrence after being in radiographic and clinical remission.

### Cjoc42 and chemotherapy studies in cancer cell lines

Cjoc42 was synthesized as previously described and confirmed by mass spectrometry (Chattopadhyay et al., 2016; D’Souza et al., 2018). Supplemental figure S1 shows HNMR spectra of Cjoc-42 used in these studies. The figure shows chemical shifts and integration values match the ones reported in the original publication of the synthesis and properties of Cjoc-42 (Chattopadhyay et al., 2016). Cjoc42 was dissolved in 100% DMSO and then diluted in 0.9% normal saline (NS) to a final concentration of 0.1% DMSO. Cisplatin (1 mg/mL; Fresenius Kabi) and Doxorubicin (2 mg/mL; Pfizer) were diluted in 0.9% NS to concentrations amenable to the treatment of cell lines. Huh6 cells were kindly gifted by Dimiter-Karl Bissig in March 2017. Cells were authenticated by the provider before transfer to the lab. Hepa1c1c7 cells were purchased from the European Collection of Authenticated Cultures (ECACC #95090613) in September 2016. The Hepa1c1c7 cells were authenticated by ECACC prior to submission. Huh6 cells were grown in Dulbecco’s Modified Eagle Medium (Fisher) and Hepa1c1c7 were grown in αMEM + GlutaMAX without nucleosides (Fisher). All media were supplemented with 10% fetal bovine serum (FBS) and 1% penicillin/streptomycin. Cells were treated with cisplatin alone, doxorubicin alone, or chemotherapy plus 1, 5, and 10 µM of Cjoc42 at a final dilution of 0.1% DMSO for 48–72 h.

TUNEL Assay (Abcam, ab66108) was conducted on untreated Huh6 cells, cells treated with cisplatin, cells treated with Cjoc42, and cells treated with cisplatin and Cjoc42 as described above. Control and treated Huh6 cells were collected after 48 h of treatment. Following treatment, cells were harvested and fixed in 1% paraformaldehyde. Cells were re-suspended in the DNA Labeling Solution consisting of Reaction Buffer, TdT Enzyme, FITC-dUTP, and ddH20 and incubated in the DNA Labeling solution for 60 min at 37°C. Cells were counterstained with Propidium Iodide/RNase A. Four to five randomly selected and non-overlapping fields were imaged on a Nikon A1 Inverted confocal microscope to observe results.

### Real-Time Quantitative Reverse Transcriptase-PCR

Total RNA was isolated from mouse and human livers using RNeasy Plus mini kit (Qiagen). cDNA was synthesized with 2 µg of total RNA using a High-Capacity cDNA Reverse Transcription Kit (Applied Biosystems). cDNA was diluted five times and subsequently used for RT-PCR assays with the TaqMan Gene Expression system (Applied Biosystems). Gene expression analysis was performed using the TaqMan universal PCR Master Mix (Applied Biosystems) according to instruction. The cycling profile was 50°C for 2 min, 95° for 10 min followed by 40 cycles of 95°C for 15 s and 60°C for 1 min as recommended by the manufacturer. TaqMan probe mixtures were purchased from ThermoFisher. The following probes were used: CUGBP1 (CELF1), Mm04279608_m1; C/EBPα, Mm01265914_s1; HNF4α, Mm01247712_m1; TP53, Mm01731290_g1; Rb1, Mm00485586_m1; cdc2(cdk1), Mm01149140_m; Caspase 6, Mm00438053_m1; ORM2, Mm04213463_g1; Human: BAX, Hs00180269_m1; TP53, Hs01034249_m1; ORM2, Hs01037491_m1; CYP3A4, Hs00604506_m1; Gankyrin (PSMD10), Hs01100439_g1. Levels of all mRNAs were normalized to β-actin.

### Protein Isolation, Western Blotting, Co-Immunoprecipitation

Nuclear and cytoplasmic extracts were prepared as previously described (Valanejad et al., 2017; Cast et al., 2018; D’Souza et al., 2018). Lysates (50 µg) were loaded on to 4–20% gradient gels (Bio-Rad) and transferred to nitrocellulose membranes (Bio-Rad). Membranes were probed with corresponding antibodies. Co-immunoprecipitations were performed using an improved True blot protocol as previously described (Valanejad et al., 2017; Cast et al., 2018; D’Souza et al., 2018; Cast et al., 2019). The following antibodies were used: CUGBP1 (Santa Cruz; sc-20003), C/EBPα (Santa Cruz; sc-61), Gank (Cell Signaling Technologies; 12985 S), HNF4α (Perseus Proteomics; PP-K9218-00), Rb (Santa Cruz; sc-50), p53 (sc-6243); β-actin (Sigma; A5316), cdc2 (Santa Cruz; sc-954). Whole gel images of all Western blots are shown in Supplemental Figures S7–12.

### Proliferation Assay

Huh6 and Hepa1c1c7 cells were seeded in 96-well plates at 5.0 × 10^4^. Images were taken at 24 h after seeding prior to treatment. Cells were treated with cisplatin and increasing concentrations of Cjoc42 (1, 5, or 10 µM) and at 48 h post-treatment, media was removed and cells were washed with 1x PBS. As per the CyQUANT Cell Proliferation Assay Kit (Invitrogen MP07026), 200 µL CyQUANT GR dye/cell lysis buffer was added to each well and incubated for 2–5 min at room temperature, protected from light. Each experiment had six to eight repeats per treatment and was repeated twice.

### Cell Culture and Cytotoxicity Assay

Huh6 and Hepa1c1c7 cells were seeded in 96-well plates at 8.0 × 10^4^. Images were taken at 24 h after seeding prior to treatment. Cells were treated with cisplatin or doxorubicin and increasing concentrations of Cjoc42 (1, 5, or 10 µM). At 48 h post-treatment, 10 µL of CCK-8 was added, as per the Cell Counting Kit-8 (Sigma-Aldrich 96992). Fluorescence was measured using a fluorescence plate reader at 450 nm. Each experiment had six to eight repeats per treatment and was repeated twice.

### Administration of Cjoc42 to Mice

Cjoc42 was dissolved in 100% DMSO. After DMSO sonication, Cjoc42 was diluted in 0.9% NS to a final DMSO concentration of 0.1%. For the mouse toxicity study, we have used 5 WT mice (males) treated with Cjoc42 and 5 WT untreated mice (males). For the studies of the stability of TSPs in mice treated with different doses of Cjoc42, we used eight animals (males) per each dose (including control DMSO-treated mice). In all studies, we have used 2-4-month-old mice. A single intraperitoneal injection was given at doses ranging from 0.1 mg/kg to 1 mg/kg (Day 1). Mice were harvested 72 h later (Day 4) with the collection of serum, liver, spleen, and kidney. Eight mice were used per group.

### Statistical Analysis

All values are presented as means ± standard error of the mean (SEM). An unpaired student t-test was applied for comparison of normally distributed data. Two way ANOVA analysis was utilized with a Bonferroni test for multiple comparisons between different time points if the *p*-value was < 0.05. Statistical significance was defined as: * = *p* < 0.05, ** = *p* < 0.01, and *** = *p* < 0.001, **** *p* < 0.0001. In instances where multiple single group comparisons were done within one experiment, the symbols “#” and “$” were utilized to differentiate the comparison group. All statistical analysis was done using GraphPad Prism 6.0.

## Results

### Elevation of Gankyrin is observed in hepatoblastoma samples with advanced disease

Our previous studies with a repository of frozen hepatoblastoma samples (51 samples) showed that Gank was 3-5-fold elevated in the majority of HBL samples (Valanejad et al., 2017; Lewis et al., 2017). However, these studies did not investigate intracellular localization of Gank in hepatoblastoma samples, nor correlate findings with clinical tumor aggressiveness. Therefore, we addressed these issues using 10 fresh samples of HBL that were available in amounts sufficient for these studies. Table 1 shows the main pathological characteristics of these patients. All patients had stage 3 or 4 disease and received pre-operative chemotherapy. Four patients had relapsed disease. Histology varied significantly and included fetal, pleomorphic, embryonal, anaplastic, and small cell undifferentiated. There was also variability in percent necrosis seen at the time of resection. The majority of patients had a reduction in alpha-fetoprotein (AFP) between diagnosis and the time of resection, although serial dilution was not done in two patients, making it impossible to know the precise percent reduction. Five patients achieved remission and remain alive. One patient relapsed twice (samples #5 and #17), and three additional patients relapsed as well. All patients with relapsed disease eventually succumbed to the disease. We performed QRT-PCR on 8/10 HBL samples and found an elevation of Gank in six patients’ tumors, while two patients’ tumors showed no elevation (Supplemental Figure S2). This number is consistent with frequency of Gank elevation previously determined by examination of a large biobank (51 HBL samples), in which we found that over 80% patients have elevated levels of Gank (D’Souza et al., 2018). In the fresh bio bank, one patient with low Gank expression had minimal necrosis, relapsed, and eventually died from the disease (Pt #7). The other patient had 50% necrosis with a good reduction in AFP and remains in remission (Pt #14). Unfortunately, we did not have pre-treated specimens from these patients to assess Gank expression at diagnosis.

**TABLE 1:** Clinical-pathological data for nine fresh samples from pediatric patients with hepatoblastoma.

Fresh sample ID #	Stage	Histology	Chemo	% Necrosis	Reduction in AFP (%)	Primary outcome	Vital Status	Gankyrin overexpression
3	3	Small cell un-differentiated	Y	30%	No	Relapse	Dead	High
5	3	Fetal pleomorphic	Y	20%	No	Relapse	Dead	High
7	4	Epithelial	Y	5–10%	Yes	Relapse	Dead	Low
8	4	RELAPSED; epithelial	Y	95%	Yes (98.3%)	Remission	Alive	High
9	3	Mixed epithelial & embryonal	Y	80%	Yes (99.8%)	Remission	Alive	High
10	4	RELAPSED; transitional cell	Y	80%	Unknown	Relapse	Dead	High
12	3	Epithelial & mesenchymal; <1% INI1-R	Y	40%	Yes	Remission	Alive	High
13	3	Epithelial, small INI1-R	Y	50%	Yes (94.6%)	Remission	Alive	High
14	3	Pleomorphic and 2% INI1-R	Y	50%	Yes (95.6%0	Remission	Alive	Low
17^a^	3	RELAPSED fetal pleomorphic, epithelial, embryonal, and small cell (INI1-R) with anaplastic features	Y	Patchy necrosis	-	Relapse	Dead	High

Gank overexpression is seen in tumor sections of the majority of patients. Abbreviations: AFP, alpha-fetoprotein; INI1-R, INI1 retained. Patient #17 and #5 are the same; sample #17 represents relapsed disease.

**FIGURE 1:**
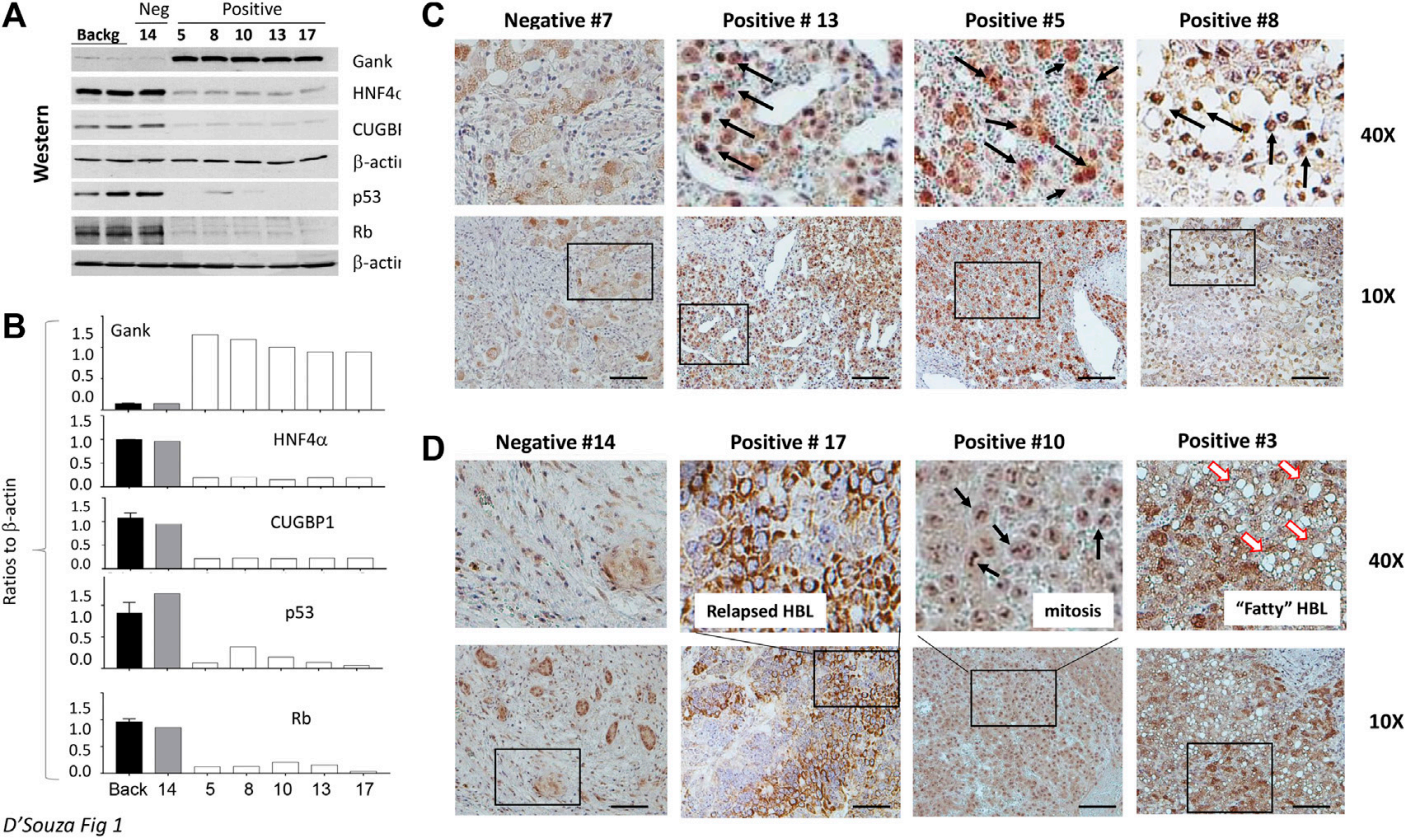
Gank-TSPs pathway is activated in the majority of hepatoblastoma samples that are characterized by chemo-resistance and by severe disease. **(A)** Western blotting analysis of expression of Gank and TSPs in nuclear extracts of background regions, Gank negative (#14), and Gank positive HBLs. **(B)** Bar graphs show quantitation of protein levels as ratios to β-actin. **(C)** Gank immunohistochemistry for patients with Gank overexpression compared to patients with no Gank overexpression. The elevated Gank is observed in nuclei of HBL patients. Arrows show nuclear Gank. **(D)** Examples of Gank staining in HBL samples with other pathological abnormalities. Arrows show mitotic figures in sample 10 and fat droplets in sample 3.

The biological activity of Gank in our HBL specimens was next assessed by measuring levels of its downstream targets i.e., tumor suppressor proteins (TSPs). Figure 1B shows the results of these studies with five HBL and three background samples. The elevation of Gank in HBL leads to a significant reduction of examined TSPs. Calculations of protein levels as ratios to β -actin revealed 3-5 fold reduction of HNF4α, CUGBP1, Rb, and p53. Levels of mRNAs were not changed significantly (data not shown) strongly suggesting that these TSPs are degraded by Gank. Background sections and a negative HBL sample (based on QRT-PCR results) had weak Gank staining; while six Gank-positive HBL showed a dramatic elevation of Gank staining across entire tumor regions (Figure 1C, D). In “Gank negative” samples, weak Gank signals are observed mainly in the cytoplasm; however, “Gank-positive” HBL samples contain Gank in nuclei and cytoplasm (Figure 1C). Since the precise nuclear quantitation of Gankyrin in immunostaining assay is difficult, we performed this calculation using Western blotting with protein extracts. We found that in background sections Gank/β-actin ratios are between 0.1–0.2; while in nuclear extracts from tumor sections, these ratios are between 1.3–1.7 (Figures 1B,C) showing a ten-fold and higher elevation of Gank in nuclei of patients with HBL. The highest total levels of Gank-immunostaining are observed in patients with relapsed HBL, in patients with highly proliferative tumors (that have high mitosis) and in patients who have steatosis (Figure 1D). This, in combination with data from 51 frozen HBL samples from a previous study (Valanejad et al., 2017), supports that a nuclear increase in Gank is commonly seen in HBL and leads to a decrease in TSPs. In regard to clinical correlation, while poor outcomes were found in patients with Gank overexpression, there were other, but rare, patients with high expression who achieved remission. Therefore, we should be careful with consideration of the levels of Gank expression as a prediction for the no response to therapy, risk for relapse, and outcome.

### Combined treatment of cancer cell lines with Cjoc42 and cisplatin leads to reduced proliferation compared to monotherapy

**FIGURE 2:**
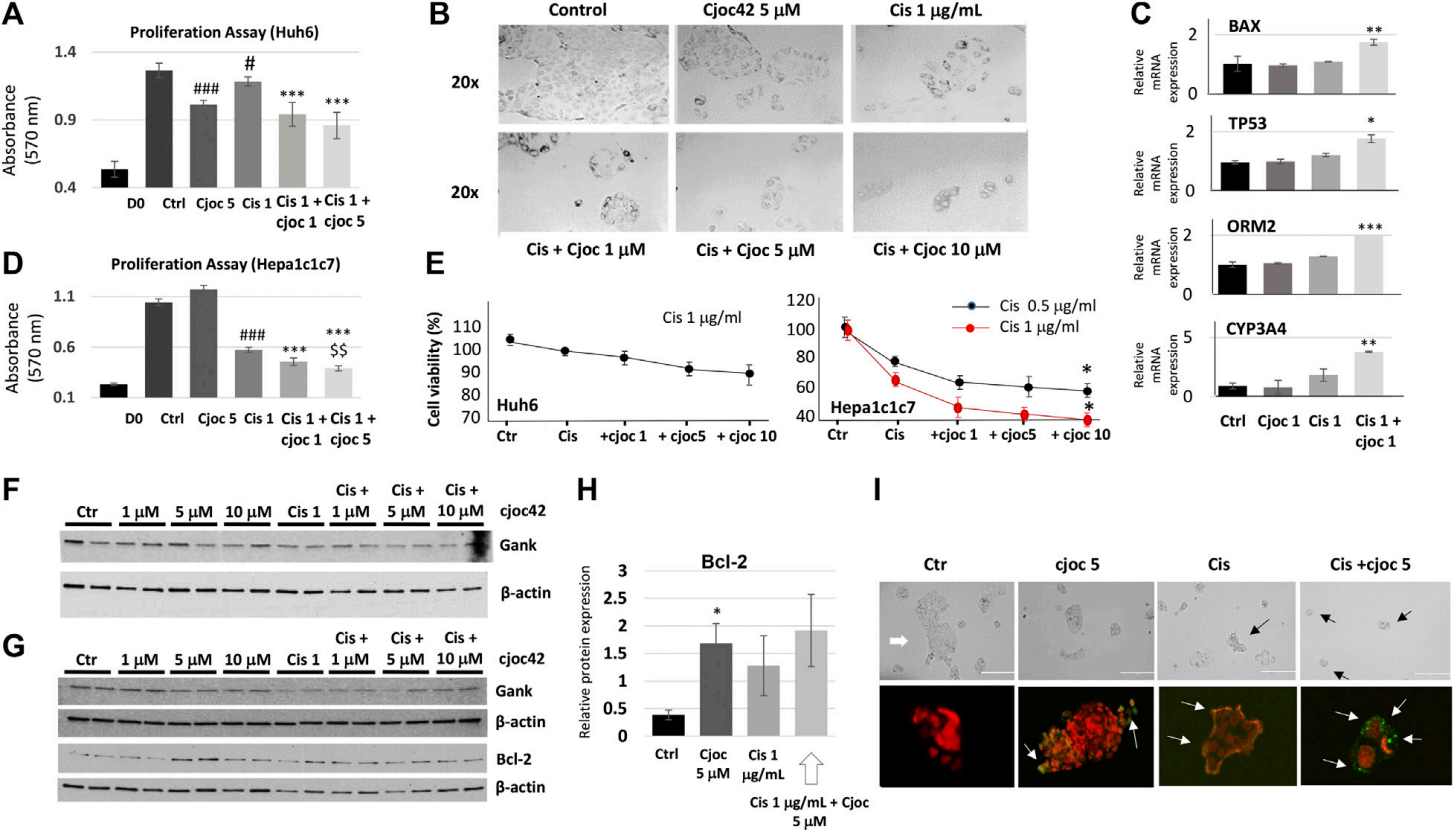
Combination treatment of cancer cell lines with Cjoc42 and cisplatin leads to stronger inhibition of proliferation compared to monotherapy. **(A)** Proliferation assay in Huh6 cells treated with Cjoc42 alone, cisplatin alone, or a combination of Cjoc42 and cisplatin. D0 shows the number of cells at the time of plating. Statistically significant reduction in proliferation is seen when comparing control to Cjoc42 5 µM (*p* < 0.0001), control to cisplatin 1 µg/mL (*p* < 0.02), cisplatin 1 µg/mL to cisplatin + Cjoc42 1 µM (*p* < 0.0001), and cisplatin 1 µg/mL to cisplatin + Cjoc42 5 µM (*p* < 0.0001). **(B)** Microscope pictures at 20x magnification show a reduction in number and cluster of Huh6 cells when treated with cisplatin and increasing concentrations of Cjoc42. **(C)** QRT-PCR data in Huh6 treated cells shows an increase in apoptotic markers Bax and TP53 with concomitant treatment as well as an increase in mature hepatocyte markers *ORM2* and *CYP3A4*. **(D)** Proliferation assay in Hepa1c1c7 cells treated with Cjoc42 alone, cisplatin alone, or a combination of Cjoc42 and cisplatin. Statistically significant reduction in proliferation is seen when comparing control to cisplatin 1 µg/mL (*p* < 0.0001), cisplatin 1 µg/mL to cisplatin + Cjoc42 1 µM (*p* < 0.0001), cisplatin 1 µg/mL to cisplatin + Cjoc42 5 µM (*p* < 0.0001), and cisplatin + Cjoc42 1 µM to cisplatin + Cjoc42 5 µM (*p* < 0.008). **(E)** Left: Cytotoxicity Assay with cisplatin 1 μg/mL and increasing concentrations of Cjoc42 (1 μM, 5 μM, and 10 µM) in Huh6 cells. Right: Cytotoxicity Assay with cisplatin 1 µg/mL and increasing concentrations of Cjoc42 (1 μM, 5 μM, and 10 µM) in Hepa1c1c7 cells. A statistically significant difference was seen in cell viability between cisplatin 0.5 µg/mL or 1 µg/mL dose alone vs. in combination with Cjoc42 10 µM (*p* < 0.01 and *p* < 0.04, respectively. **(F)** Western blot analysis of Huh6 cells treated with cisplatin 1 µg/mL and increasing concentrations of Cjoc42. **(G)** Western blot analysis of Hepa1c1c7 cells treated with cisplatin 1 µg/mL and increasing concentrations of Cjoc42. A statistically significant difference was seen in cell viability between cisplatin 0.5 µg/mL or 1 µg/mL dose alone vs. in combination with Cjoc42 10 µM (*p* < 0.01 and *p* < 0.04, respectively). **(H)** A statistically significant difference in Bcl2 is seen comparing control cells to Cjoc42 5 µM (*p* < 0.03). **(I)** TUNEL assay of Huh6 cells treated with Cjoc42 (5 µM), cisplatin (1 µg/mL), and with combination cisplatin plus Cjoc42. The upper panel shows typical microscope pictures of the same cells before staining. White arrow shows a big colony of untreated Huh6 cells, black arrows show small colonies in treated cells. Bottom images show the merge of the staining with Propidium Iodide/RNase A (red) and FITC-dUTP (green).

Given the increased Gank in patients with HBL (Figure 1), we asked if the inhibition of Gank might reduce the chemoresistance of cancer cells. Huh6 and Hepa1c1c7 cells were treated with DMSO (Control), Cjoc42 5 μM, Cisplatin 1 µg/mL, or with a combination of Cisplatin 1 µg/mL and Cjoc42 1 µM or 5 µM. We found a statistically significant reduction in proliferation of Huh6 cells when comparing cisplatin 1 µg/mL to cisplatin + Cjoc42 1 µM or cisplatin + Cjoc42 5 µM (Figure 2A). Figure 2B presents typical images of the treated cells and illustrates the reduction in the number and size of clusters of Huh6 cells. QRT-PCR demonstrated a statistically significant increase in apoptotic proteins Bax and TP53. Combination treatment also lead to an increase in mature hepatocyte markers *ORM2* and *CYP3A4* (Figure 2C). Hepa1c1c7 cells treated with similar combinations of Cjoc42 and cisplatin revealed a stronger effect on proliferation than by either agent alone (Figure 2D). Overall, combination treatment with cjoc42 and cisplatin resulted in a strong inhibition of proliferation compared to cisplatin alone.

### The combination therapy of cisplatin and Cjoc42 resulted in the enhanced cytotoxicity

Next, we examined the effects of the combined treatments on cytotoxicity. Because the differences seen by proliferation assay were between cisplatin and cisplatin in combination with cjoc42, we focused our cytotoxicity analysis there. We found a modest reduction in cell viability in Huh6 cells with the addition of Cjoc42 compared to cisplatin alone, which was not statistically significant (Figure 2E). A statistically significant increase in cytotoxicity was evident in Hepa1c1c7 cells treated with cisplatin 1 µg/mL and Cjoc42 10 µM compared to cisplatin 1 µg/mL alone (Figure 2E). The difference in the sensitivity of Huh6 and Hepa1c1c7 cells on combination treatments might be related to different origin of these cancer cells. Levels of Gank were not affected by Cjoc42 (Figure 2F). Of note, minimal cytotoxicity was seen in Huh6 cells or Hepa1c1c7 cells treated with Cjoc42 alone (Supplemental Figure S3). Western blotting analysis of Hepa1c1c7 cells showed a statistically significant increase in Bcl2 in cells treated with Cjoc42 5 µM compared to control cells (Figure 2G,H). We found a similar increase of Bcl2 between Hepa1c1c7 cells treated with cisplatin with Cjoc42 5 µM compared to cisplatin alone; however, this was not statistically different. We next performed a TUNEL assay. The Huh6 cells grow as colonies with increased numbers of cells per colony and by forming clusters. We found apoptotic cells within clusters of each experimental group. The untreated Huh6 cells have a low, but detectable level of apoptotic cells, while treatment with Cjoc42 and cisplatin alone increase numbers of the apoptotic cells in the colonies. In cells with combined treatment, the number of apoptotic cells was increased compared to either agent alone (Figure 2I). Additional images of the TUNEL assay can be found in Supplemental Figures S4A–D. A significant portion of apoptotic Huh6 cells were detached from the plate, making precise quantitation difficult. Overall, these experiments suggest that combination treatment of liver cancer cell lines with cjoc42 and cisplatin leads to improved cytotoxicity by increased apoptosis.

### Combination therapy of doxorubicin and Cjoc42 also shows enhanced cytotoxicity

**FIGURE 3:**
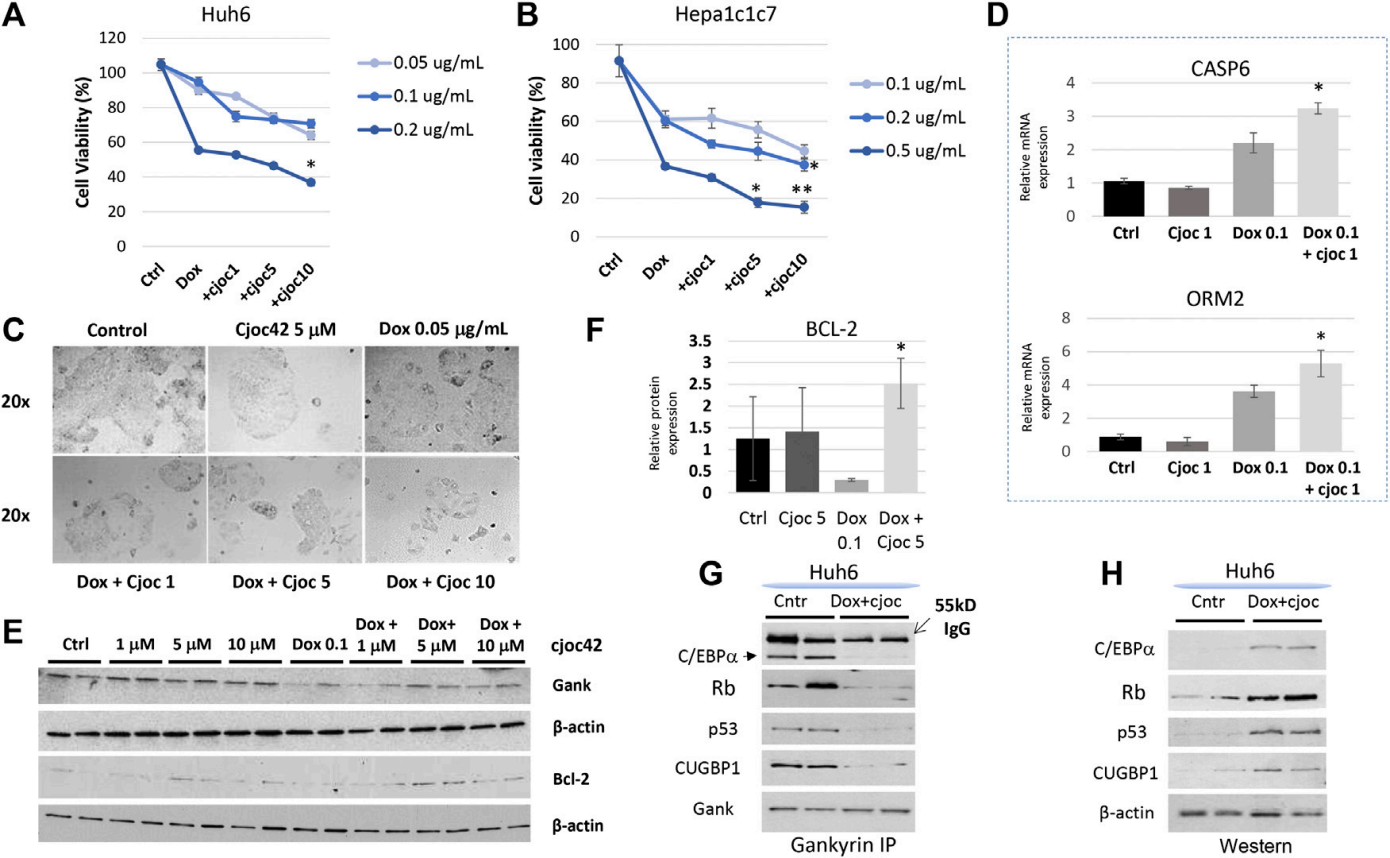
A combination therapy of doxorubicin and Cjoc42 enhances cytotoxicity. **(A)** Cytotoxicity Assay with doxorubicin (0.05, 0.1, and 0.2 µg/mL) and increasing concentrations of Cjoc42 (1, 5, and 10 µM) in Huh6 cells. **(B)** Cytotoxicity Assay with doxorubicin (0.1, 0.2, and 0.5 µg/mL) and increasing concentrations of Cjoc42 (1, 5, and 10 µM) in Hepa1c1c7 cells. **(C)** Microscope pictures at 20x showing reduction in size and number of clustered Huh6 cells when treated with doxorubicin 0.05 µg/mL and increasing concentrations of Cjoc42 compared to Cjoc42 or doxorubicin alone. **(D)** QRT-PCR data with Hepa1c1c7 cells treated with 1 µM Cjoc42 alone, Doxorubicin 0.1 µg/mL alone, or a combination of both. There was a statistically significant increase in expression of the apoptotic marker Casp6 with combination treatment (*p* < 0.05) and the mature hepatocyte marker ORM2 (*p* < 0.05). **(E)** Western blotting in Huh6 cells treated with Cjoc42 alone, doxorubicin 0.05 µg/mL, or a combination of doxorubicin and Cjoc42. **(F)** Bcl2 expression was calculated as a ratio to β-actin. Bcl2 is significantly higher in cells treated with doxorubicin 0.1 µg/mL and Cjoc42 5 µM compared to doxorubicin 0.05 µg/mL alone (*p* < 0.03). **(G)** Co-IP studies. Gank was immunoprecipitated from untreated Huh6 and from Huh6 cells treated with doxorubicin (0.05 µg/mL) and cjoc42 (5 µM). Tumor suppressor proteins were examined in these IPs. **(H)** Western blotting shows levels of tumor suppressor proteins in nuclear extracts of untreated and doxorubicin + cjoc42 treated Huh6 cells.

Since another chemotherapeutic agent, doxorubicin, is frequently used for treatments of HBL patients, we next examined if a combination of Cjoc42 with doxorubicin might enhance cytotoxicity. In Huh6 and Hepa1c1c7 cells, a statistically significant reduction in cell viability was observed with combination therapy using higher doses of Cjoc42 (Figure 3A,B, respectively). Microscope pictures of both Huh6 (Figure 3C) and Hepa1c1c7 (Supplemental Figure S5) cells show a visible reduction in the number of cells that are treated with doxorubicin in combination with increasing concentrations of Cjoc42. Apoptotic marker Caspase six and mature hepatocyte marker ORM2 was increased in Hepa1c1c7 cells treated with doxorubicin + Cjoc42 1 µM compared to doxorubicin alone by QRT-PCR (Figure 3D). Additionally, Western blotting showed that there was a statistically significant increase in BCL-2 expression in Hepa1c1c7 cells treated with doxorubicin 0.1 µg/mL + Cjoc42 5 µM compared to treatments with doxorubicin alone (Figure 3E,F). Since Gank triggers the degradation of tumor suppressor proteins in patients with HBL (Figure 1B), we examined if this activity of Gank is inhibited in Huh6 cells treated by doxorubicin and cjoc42. By co-immunoprecipitation (Co-IP), we observed that combination treatment reduces the interactions of Gank with C/EBPα, Rb, p53, and CUGBP1 (Figure 3G) and increases levels of TSPs (Figure 3H) compared to no treatment. Altogether, this suggests that combining Cjoc42 and doxorubicin leads to reduced cell viability and increased apoptosis of liver cancer cells due to inhibition of the Gank-dependent degradation of TSPs and elevation of their levels.

### Cjoc42 is tolerable in mice

**FIGURE 4:**
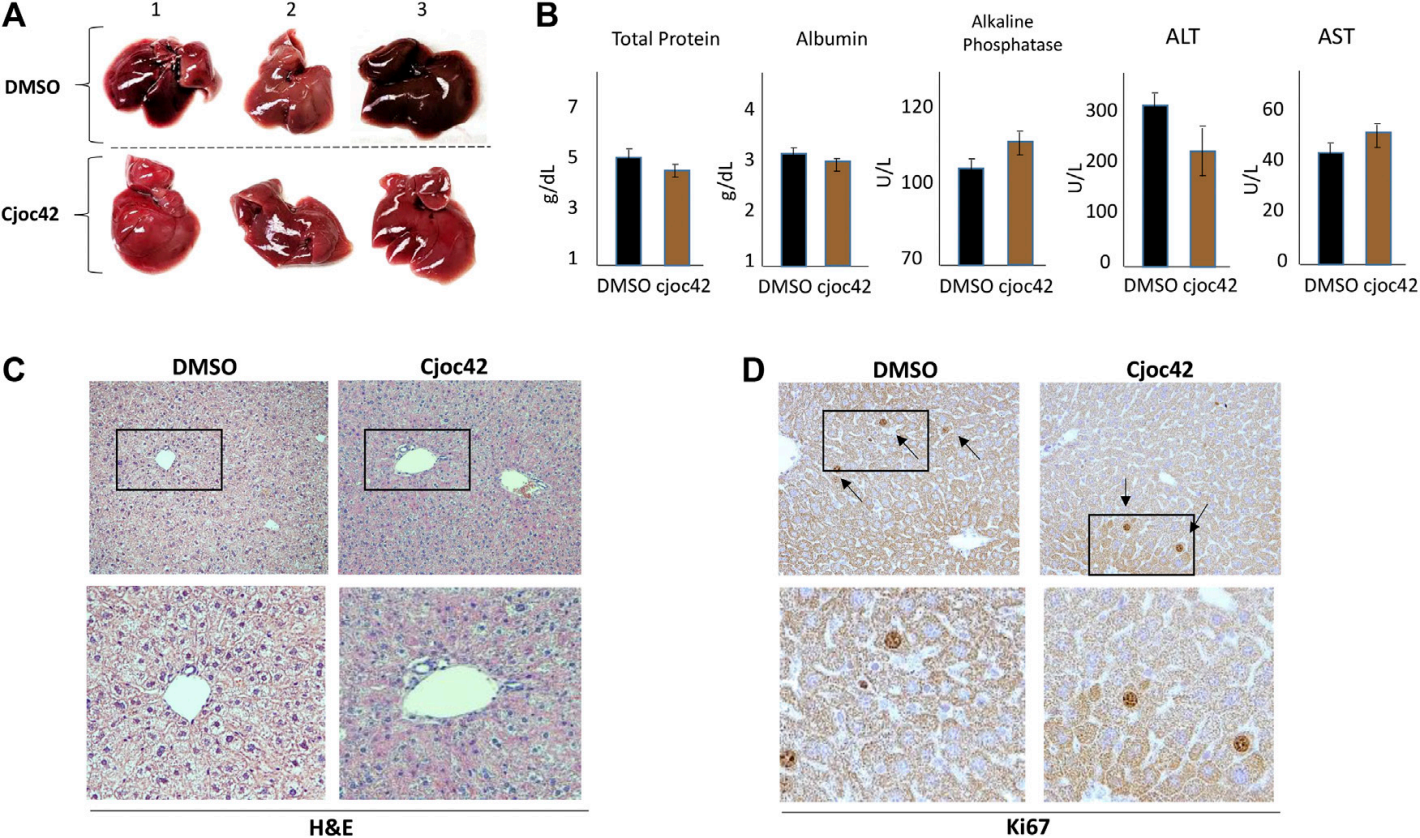
Toxicity study in mice shows that Cjoc42 is safe to administer. **(A)** Gross liver examination of three mice treated with DMSO and three mice treated with Cjoc42 1 mg/kg. No morphological differences were noted. **(B)** Serum hepatic parameters were analyzed with no differences seen between DMSO and Cjoc42 1 mg/kg treated mice. The serum was collected and analyzed on five mice in each group. **(C, D)** Histological examination of the livers of DMSO and Cjoc42 1 mg/kg treated mice at 10x magnification. Bottom images show images of regions of livers (boxed) under higher magnification. No differences were seen in H&E **(C)** and Ki67 **(D)** staining.

Although Cjoc42 had been examined in cultured cells, no prior studies had been done in mice. Therefore, we injected five wild type mice with a single intraperitoneal dose of Cjoc42 of 0.1 mg/kg, 0.25 mg/kg, 0.5 mg/kg, or 1 mg/kg. Mice were harvested 72 h later. All mice remained visibly healthy and mobile with stable weight (Supplemental Figures 6A,4B). Additionally, there were no differences in liver/body ratio, spleen weight, or kidney weight in all examined mice (N = 5, Supplemental figure S6C). Figure 4A demonstrates a normal gross examination of livers of mice treated with DMSO compared to Cjoc42 1 mg/kg. The liver function panel was also similar between both groups (Figure 4B). Immunohistochemistry analysis found no differences detected by H&E and Ki67 (Figure 4C,D). These studies revealed that the administration of Cjoc42 in a dose up to 1 mg/kg bodyweight does not cause toxic effects.

### Cjoc42 stabilizes tumor suppressor proteins in mice by inhibition of their interactions with Gank

**FIGURE 5:**
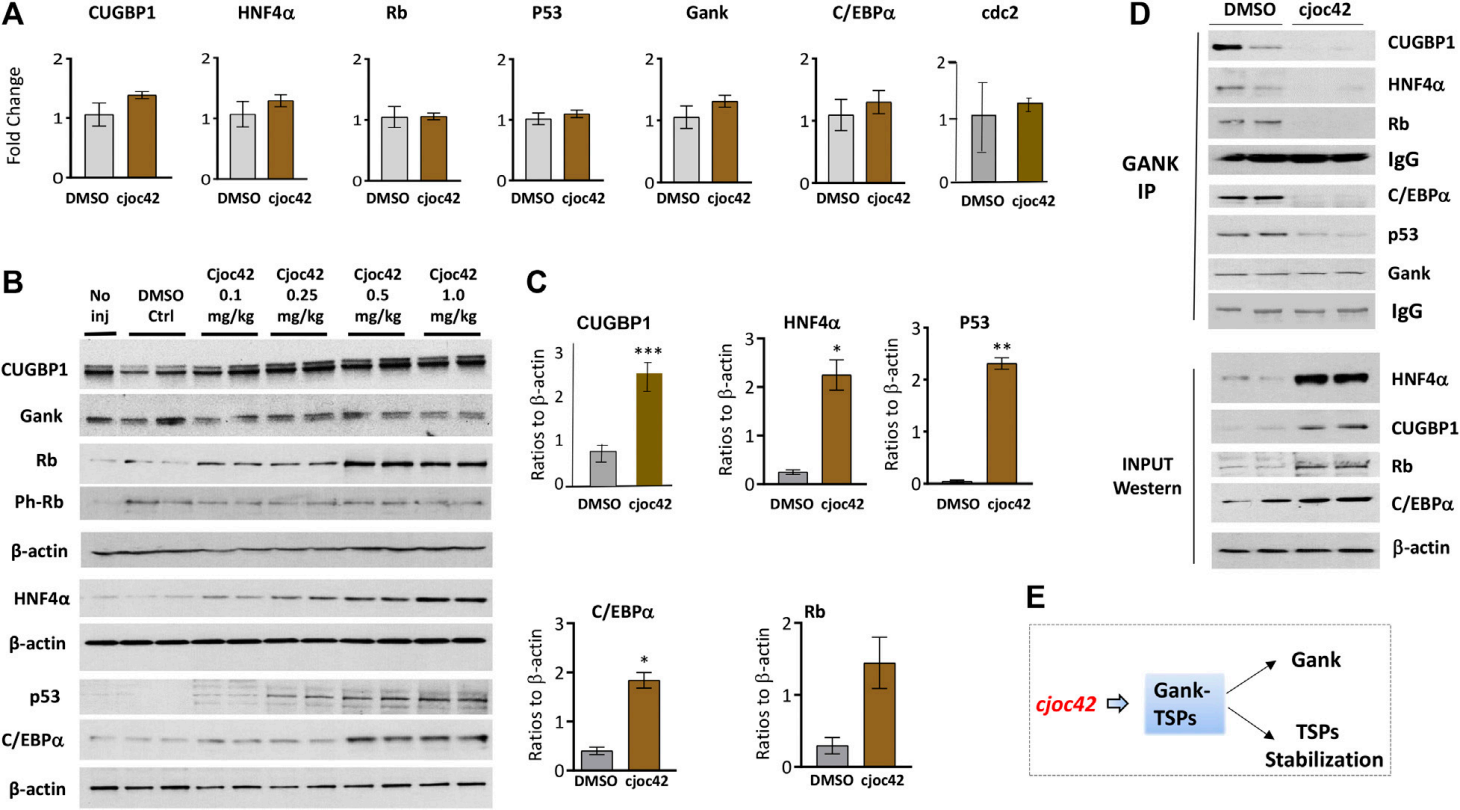
Treatments of WT mice with Cjoc42 inhibit interactions of Gank with TSPs and increase levels of the TSPs***.* (A)** QRT-PCR analysis shows no difference in the mRNA levels of TSPs CUGBP1, HNF4α, Rb, p53, C/EBPα, Gank, and cell cycle marker cdc2 between DMSO and Cjoc42 treated mice. **(B)** Western blot analysis of TSPs and Gank in mice with no injection, DMSO, or varying doses of Cjoc42 (0.1, 0.25, 0.5, and 1 mg/kg). **(C)** Quantitation of western blots for p53 (*p* < 0.0024), C/EBPα (*p* < 0.014), Rb (*p* > 0.05), CUGBP1 (*p* < 0.004) and HNF4α (*p* < 0.01) comparing DMSO to Cjoc42 1 mg/kg dose. **(D)** Co-IP studies with DMSO treated and Cjoc42 (1 mg/kg) treated mice. The upper image shows the results of Co-IP. The bottom image shows input (loading of proteins). IgG shows signals of heavy chains of immunoglobulins G added to the IP reactions. **(E)** The diagram summarizes the results of Cjoc42 treatments on the stability of TSPs in livers.

Next, we examined the biological activities of Cjoc42 in livers of injected mice. Since one of the key oncogenic activities of Gank is triggering degradation of tumor suppressor proteins C/EBPα, HNF4α, p53, Rb, and CUGBP1 (Valanejad et al., 2017; D’Souza et al., 2018), we examined if Cjoc42 will inhibit this activity of Gank. QRT-PCR demonstrated no significant differences in levels of CUGBP1, Rb, p53, C/EBPα, and HNF4α mRNAs. The levels of Gank mRNA and mRNA of proliferation marker cdc2 were also not changed (Figure 5A). However, we found a statistically significant increase in protein levels of CUGBP1, Rb, C/EBPα, p53, and HNF4α in the livers of mice treated with Cjoc42 at concentrations of 0.5 and 1 mg/kg compared to mice treated with DMSO. Note that all these TSPs are expressed in livers and are only partially degraded by Gank in quiescent livers. The significant portions of each TSP are resistant to Gank degradation due to post-translational modifications. For example, de-ph-S302-CUGBP1 is degraded by Gank, but ph-S302-CUGBP1 isoform does not interact with Gank and it is resistant to degradation (Lewis et al., 2017). Normalized to β-actin, p53, Rb, C/EBPα, HNF4α, and CUGBP1 were elevated 3-4 fold (Figure 5B,C). Interestingly, despite the elevation of total levels of Rb, the ph-780-Rb form was not increased in Cjoc42-treated mice compared to control DMSO-treated mice. Since non-phosphorylated Rb is a stronger tumor suppressor, the Cjoc42-mediated elevation of the de-ph-S780-Rb suggests that the liver might be more resistant to the development of cancer in Cjoc42-treated mice. Co-IP demonstrated reduced interactions of Gank with Rb, C/EBPα, CUGBP1, and HNF4α in Cjoc42 treated livers. In summary, we found that a single intraperitoneal injection of Cjoc42 is sufficient to block interactions of Gank with TSPs and to increase the levels of these proteins (Figure 5E).

## Discussion

One common molecular feature of the development of pediatric and adult liver cancers is the elimination of TSPs. Growing evidence shows that proteolytic degradation of TSPs is one of the key pathways of this elimination (Bashir and Pagano, 2003; Hanahan and Weiberg, 2011; Jiang et al., 2013; Valanejad et al., 2017). The important questions in the development of Gank-based drugs for HBL are: 1) do patients with hepatoblastoma have activation of the Gank-TSPs pathway; 2) is the activation of this pathway in cancer a critical part of HBL pathophysiology; and 3) will the potential inhibition of this pathway by Cjoc42 have a beneficial outcome? Our work addressed these questions by investigations of 10 fresh HBL samples, which were collected in CCHMC within the last two years, and established cancer cell lines. We found that an elevation of Gank and almost complete degradation of the TSPs are observed in the majority of examined patient tumors. This is also consistent with what was found in 51 frozen HBL samples within our biorepository, which has previously been published (Valanejad et al., 2017). The elevation of protein levels of Gank is higher than the elevation of Gank mRNA (Supplemental Figure S1; Figure 1). This difference is likely to be associated with a very long half-life of the Gank protein which is around 800 h in primary hepatocytes (Mathieson et al., 2018). We also examined the utility of Gank immunostaining and demonstrated increased nuclear expression of Gank in the majority of samples. We did not find a clear correlation between Gank overexpression and tumor aggressiveness or outcome; however, this is likely due to small patient numbers.

For cytotoxicity studies, we selected two commonly used and well-investigated liver cancer cell lines, Huh6 (human hepatoblastoma) and Hepa1c1c7 (mouse HCC). Using cell proliferation assays and colony size by microscopy, we found that the combination of cjoc42 with cisplatin, as well as with doxorubicin, inhibits proliferation of cells more than chemotherapy alone (Figures 2, 3). This is likely in part due to rescue of tumor suppressor proteins seen with Cjoc42 treated cells (Figure 3G,H) as this provides a different mechanism for cell death than traditional cytotoxic chemotherapy. Mechanistically, the activation of the markers of apoptosis Bax, Bcl2 and TP53, as well as the TUNEL assay strongly suggest that the combined treatment lead to apoptotic death of the cancer cells and the selection of the “healthy”, hepatocyte-like cells since we found an increase of the mature hepatocyte markers *ORM2* and *CYP3A4* in cells with combined treatments. We have also found that there is a variability in cytotoxicity seen in Huh6 and Hepa1c1c7 cells. Although, this might be related to different origins of these cancer cells, our studies showed that overall, combined therapy enhances the cytotoxicity of chemotherapy. Examination of the ability of Gank to degrade tumor suppressors revealed that the combined doxorubicin + cjoc42 treatments of cancer cells stabilize tumor suppressors (Figure 3G,H). The doxorubicin + cjoc42-mediated increase of tumor suppressors might be involved in elevation of mature hepatocytes markers. Taking together our results, we think that cjoc42 might be considered as a therapeutic strategy to increase the cytotoxic effects of cisplatin and doxorubicin.

Since cjoc42 is a new compound with pharmacological potential, until now it had only been investigated in tissue culture systems. The previous studies showed that tumor suppressor proteins C/EBPα, HNF4α, Rb, p53 and CUGBP1 are abundant in quiescent liver and that their levels are controlled by Gank-dependent degradation (Lewis et al., 2017; Valanejad et al., 2017; Matheison et al., 2018; Valanejad et al., 2018). We found that, at doses 0.5–1 mg/kg, Cjoc42 inhibits the ability of Gank to degrade hepatic tumor suppressor proteins. This data also supports that the drug reached its target (i.e. the liver) and toxicity analysis demonstrated it was well tolerated. Our studies were focused on the Cjoc42-mediated change of Gank activities toward tumor suppressors; however, Gank also affects several additional pathways of liver cancer including NF-kB pathway (Li et al., 2018), β-catenin pathway (He et al., 2016; Liu et al., 2019). Gank also stabilizes stem cell markers such as Oct4 (Qian et al., 2012). We think that these additional pathways might be potentially inhibited by Cjoc42. Taking together our *in vivo* studies and these potential pathways, we believe that further investigations of Cjoc42 as a therapeutic agent for HBL through inhibition of Gank are warranted.

Given the frequency of Gank overexpression in HBL and clear evidence of its carcinogenic effects, Gank inhibition therapy might be considered for future clinical trials. It is important to note that as a single agent, Cjoc42 was not cytotoxic. However, use of Cjoc42 in combination with cisplatin or doxorubicin, which are frequently used in HBL, enhanced the cytotoxicity of chemotherapy. This exciting finding supports the consideration of combination treatment with a Gank inhibitor and chemotherapy as a way to enhance chemosensitivity in relapsed, refractory HBL. It is also important to note that recently the second generation of cjoc42-based inhibitors of Gank showed a stronger inhibition of cell proliferation (Kanabar et al., 2020) and might be considered for the further pre-clinical studies both as a single agent and in combination with chemotherapy.

## Data Availability

The original contributions presented in the study are included in the article/Supplementary Material, further inquiries can be directed to the corresponding authors.
