# Supplementary material for: Small Molecule Cjoc42 Improves Chemo-Sensitivity and Increases Levels of Tumor Suppressor Proteins in Hepatoblastoma Cells and in Mice by Inhibiting Oncogene Gankyrin
Source: Front Pharmacol. 2021 Mar 4;12:580722. doi: 10.3389/fphar.2021.580722 (PMC7969996; doi:10.3389/fphar.2021.580722)
Supplement: Supplementary file 1 [file datasheet1.pdf]

Supplemental Materials For The Manuscript By

Amber M. D’Souza, Ashley Cast, Meenasri Kumbaji, Maria Rivas, Ruhi Gulati,
Michael Johnston, David Smithrud, James Geller, Nikolai Timchenko

TITLE:
Small Molecule Cjoc42 Improves Chemo-sensitivity and Increases Levels of Tumor Suppressor Proteins in Hepatoblasoma Cells and in Mice by Inhibiting Oncogene Gankyrin.

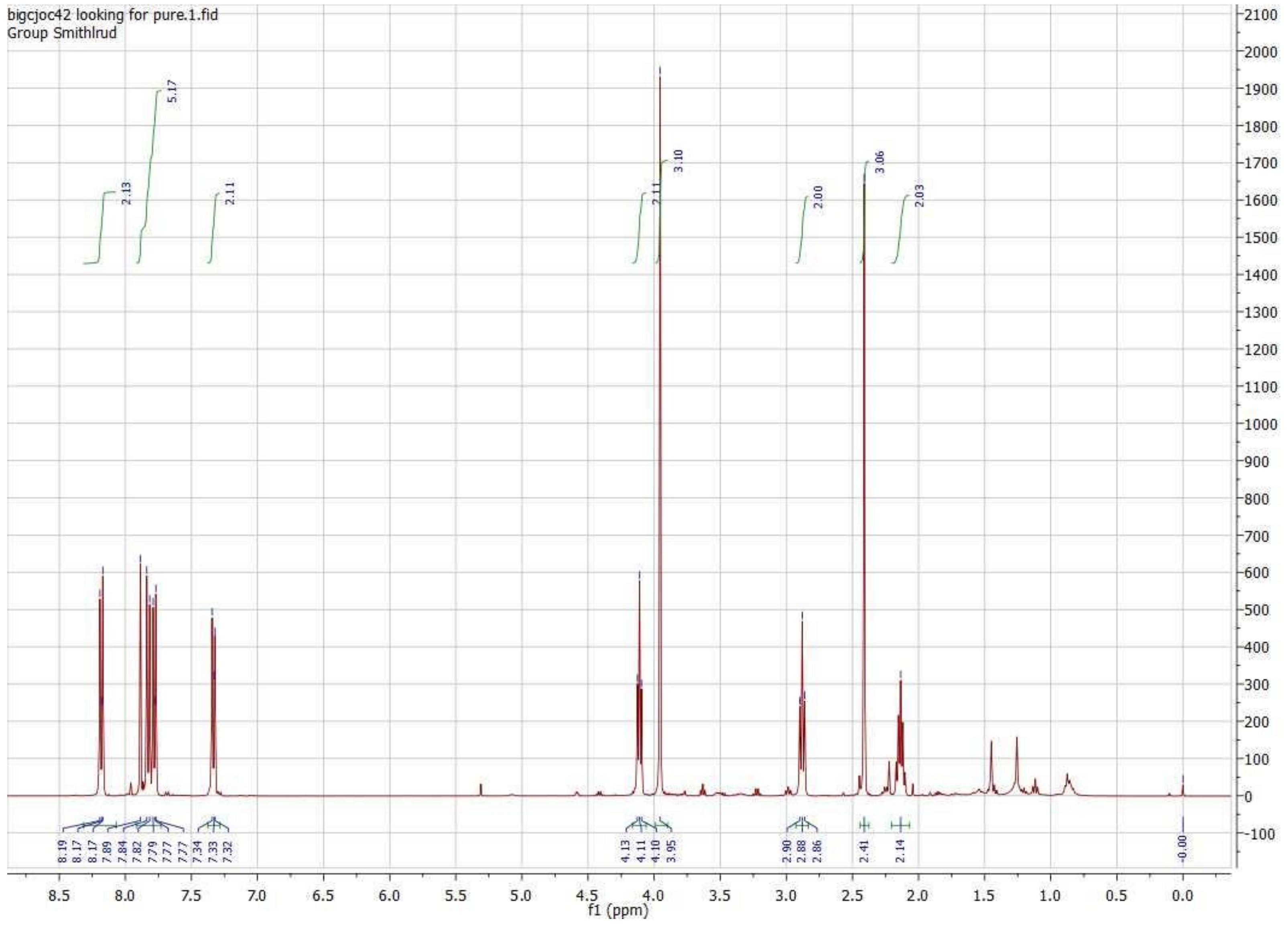

D'Souza, Supplemental Figure 1. HNMR spectra of cjoc-42 used in these studies. The figure shows chemical shifts and integration values match the ones reported in the original publication of the synthesis and properties of cjoc-42 (Chattopadhyay et al., 2016).

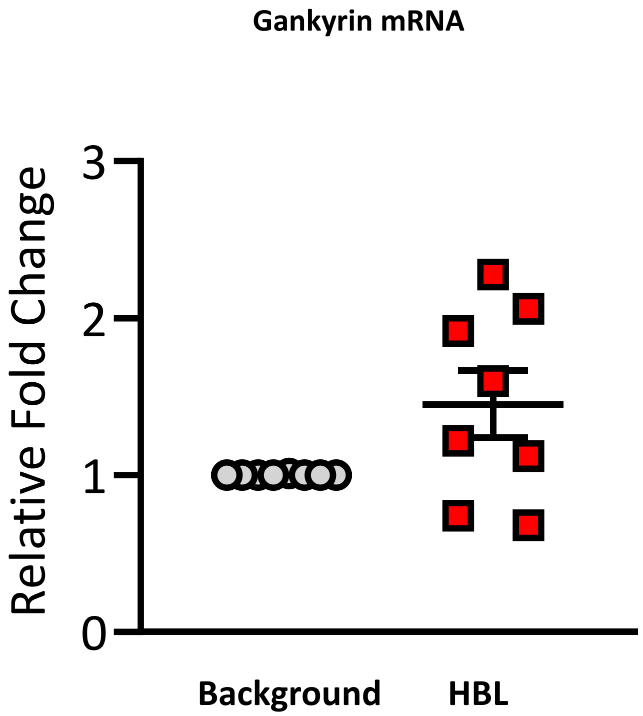

D'Souza, Supplemental Figure 2. QRT-PCR shows levels of Gank mRNA in fresh HBL samples.

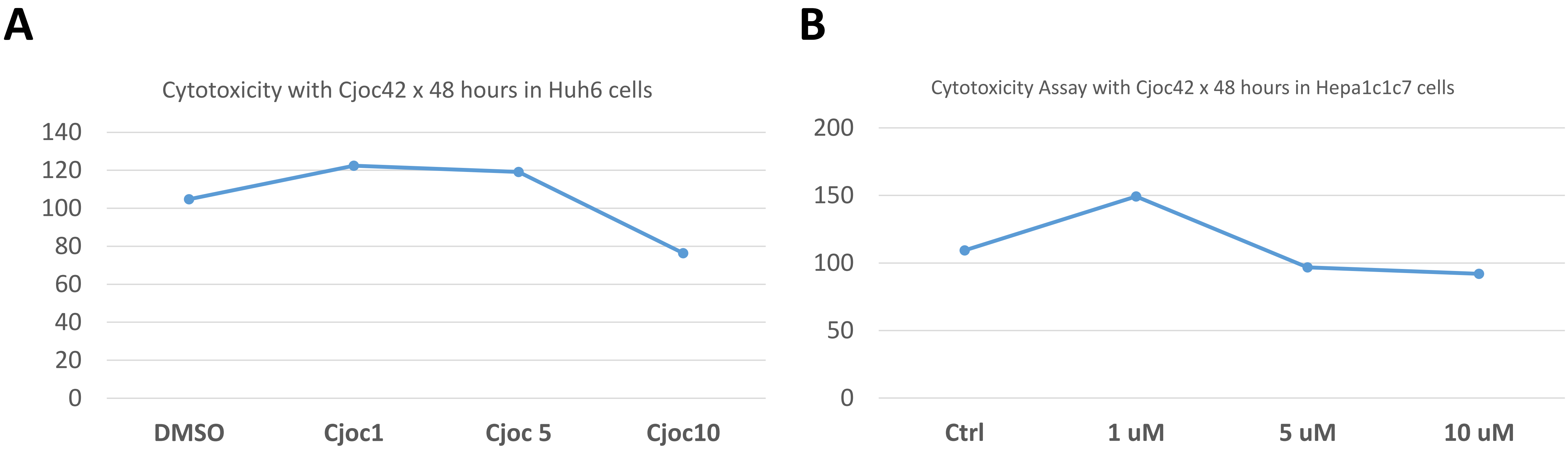

D'Souza, Supplemental Figure 3. **Cytotoxicity in Huh6 and Hepa1c1c7 cells treated with cjoc42 alone. (A)** Cytotoxicity assay in Huh6 cells treated with cjoc42 alone compared to DMSO control showed a modest decrease in cell viability at the 10 µM dose, but not statistically significant ($p > 0.14$). **(B)** Cytotoxicity assay in Huh6 cells treated with cjoc42 alone compared to DMSO control showed no difference in cell viability.

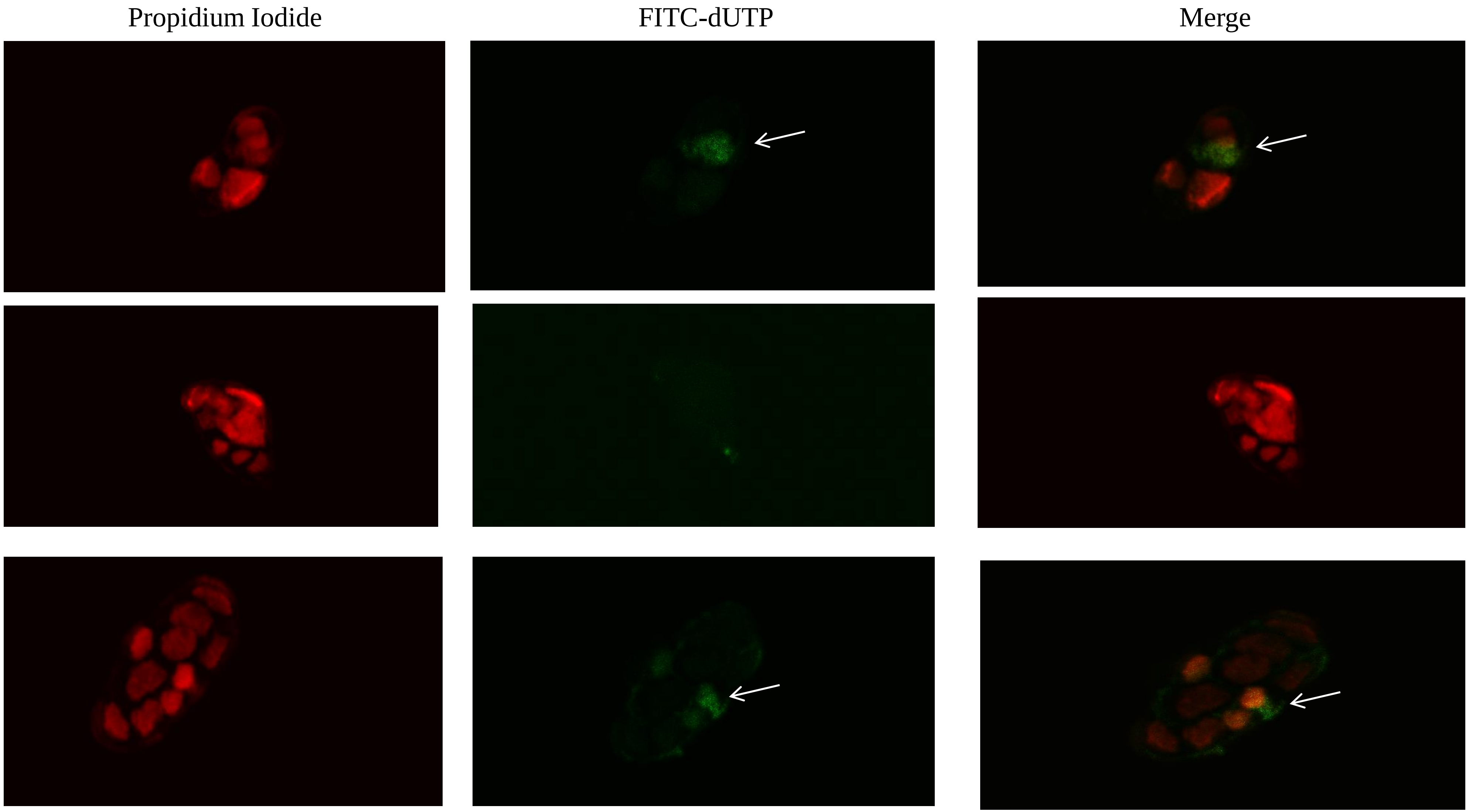

D'Souza, *Supplemental Figure 4a*. *Examples of TUNEL assay in control, untreated Huh6 cells. Arrows show apoptotic cells within clusters. Supplements to Fig 2I.*

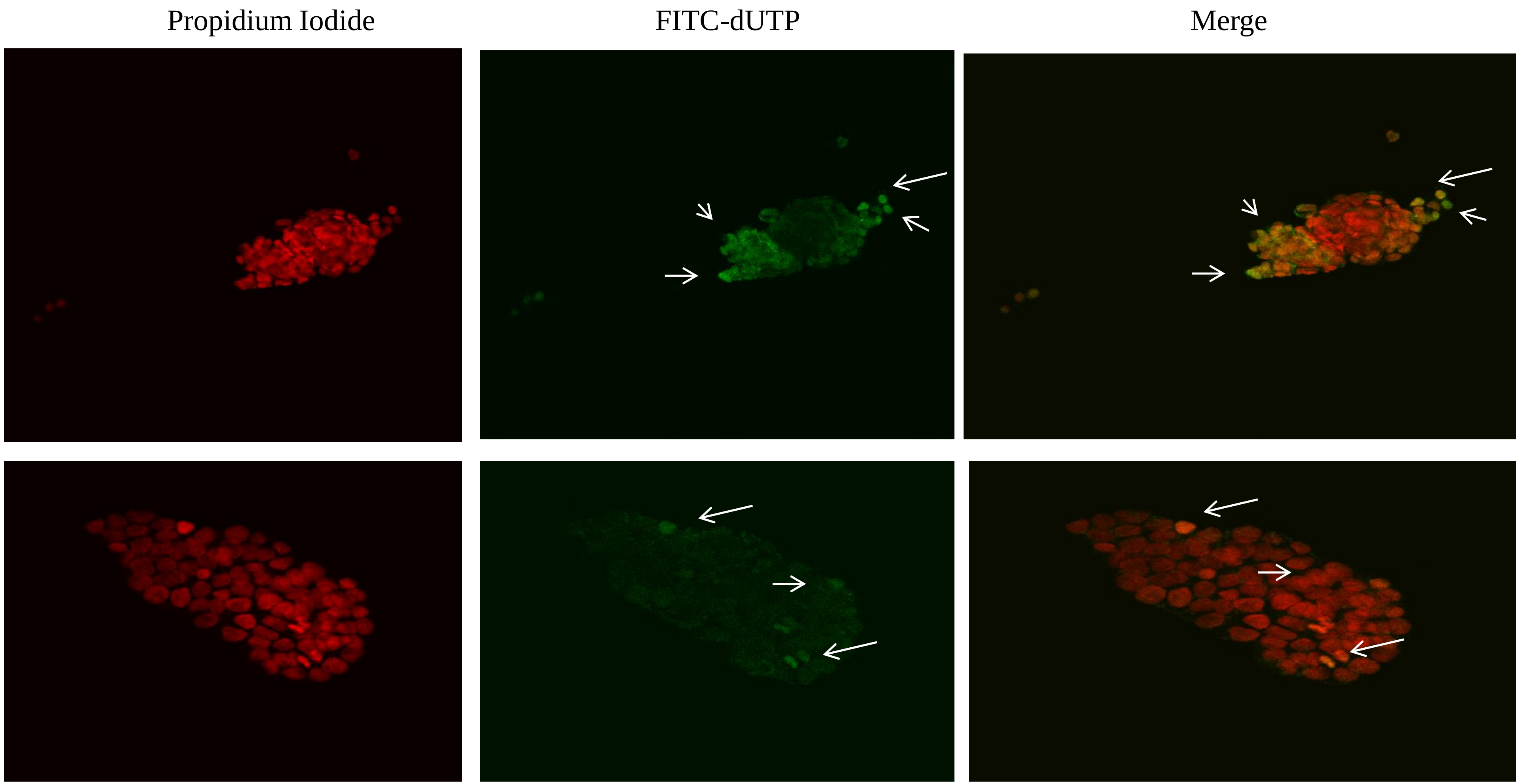

D'Souza, *Supplemental Figure 4b*. *Examples of TUNEL assay in cjoc42-treated Huh6 cells. Arrows show apoptotic cells within clusters. Supplements to Fig 2I*

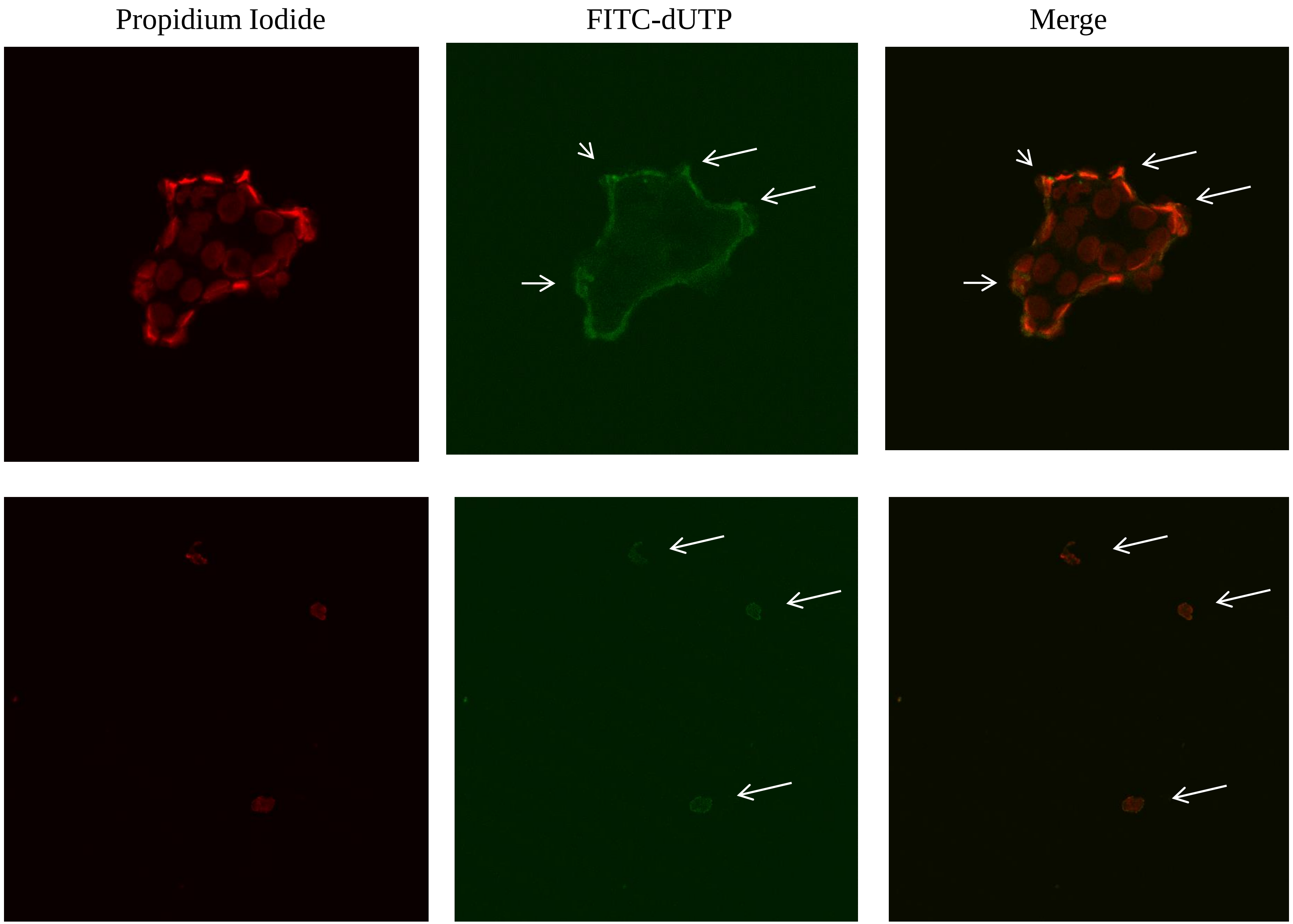

D'Souza, *Supplemental Figure 4c*. *Examples of TUNEL assay in cisplatin-treated Huh6 cells. Arrows show apoptotic cells within clusters. Supplements to Fig 2I.*

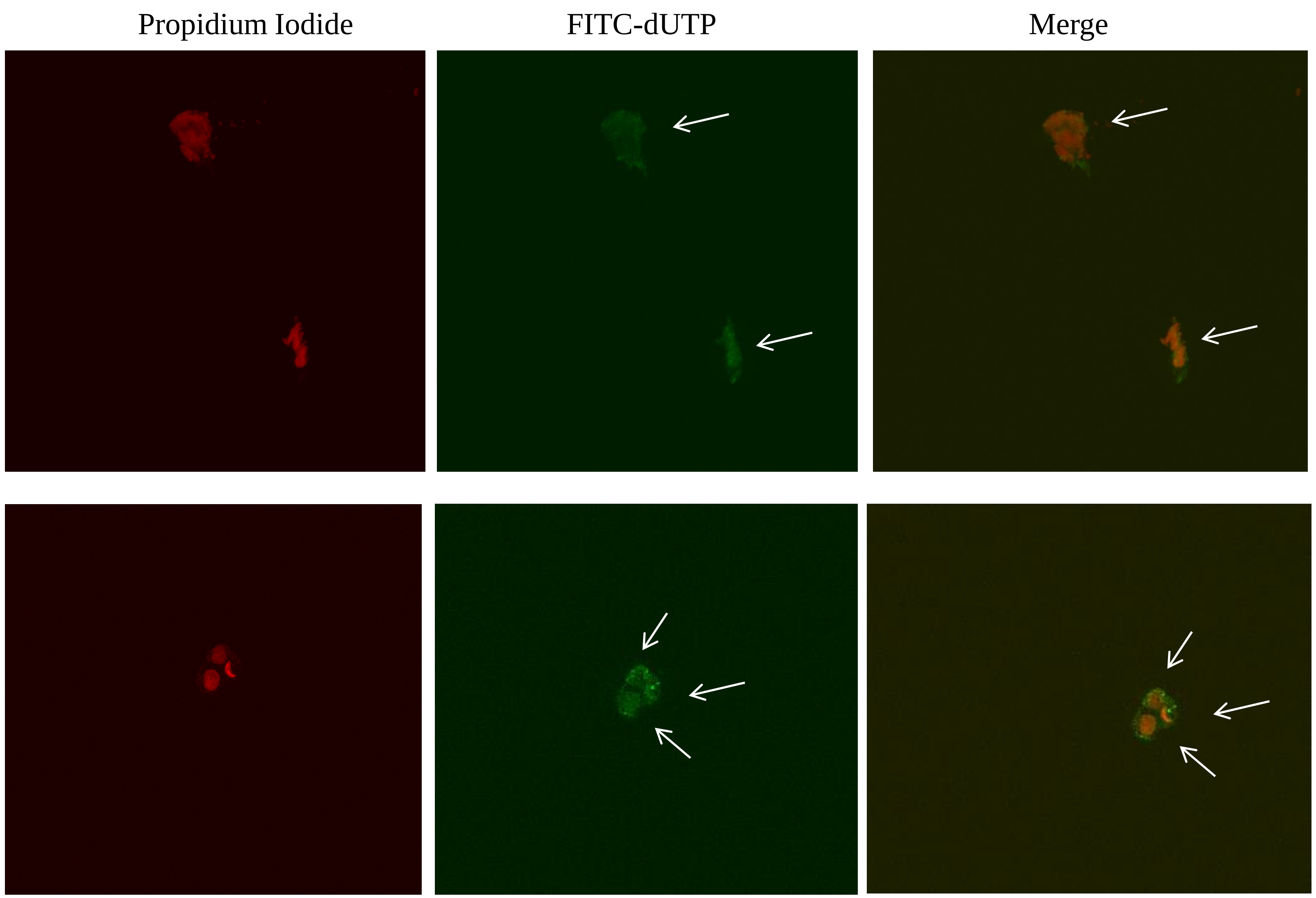

D'Souza, *Supplemental Figure 4d*. *Examples of TUNEL assay in cjoc42 + cisplatin treated Huh6 cells. Arrows show apoptotic cells within clusters. Supplements to Fig 2I.*

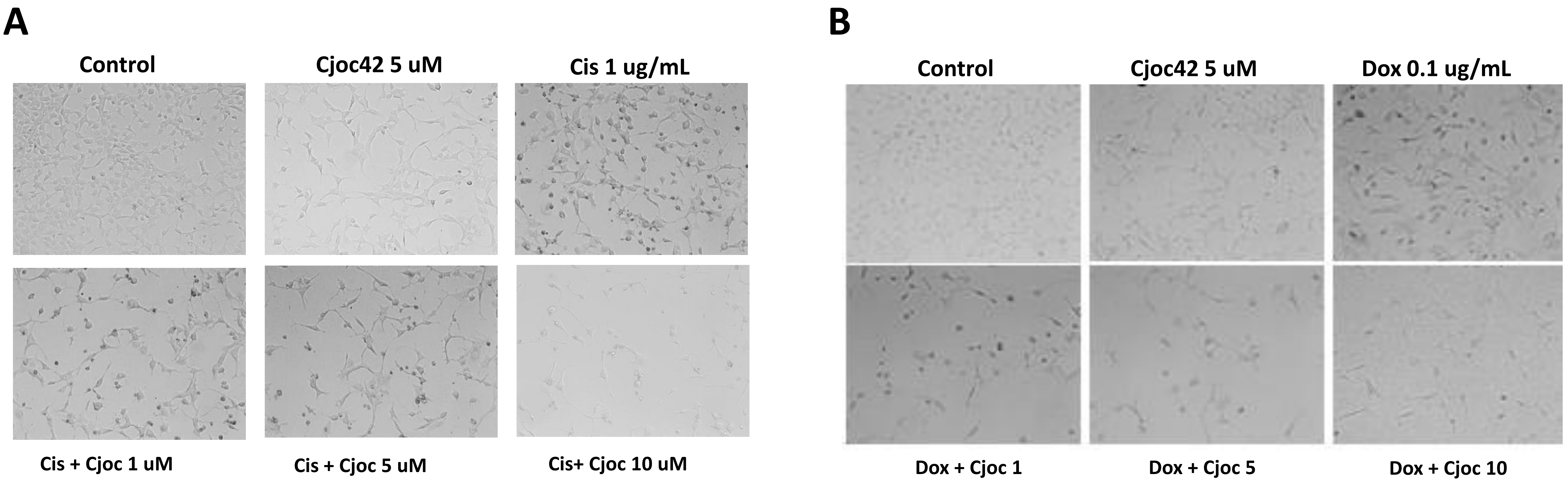

D'Souza, Supplemental Figure 5.

A) Microscope pictures at 20x magnification show reduction in number of Hepa1c1c7 cells when treated with ***cisplatin*** and increasing concentrations of Cjoc42.

B) Microscope pictures at 20x showing reduction in size and number of clustered Hepac1c7 cells when treated with ***doxorubicin*** 0.1 mg/mL and increasing concentrations of Cjoc42 compared to cjoc42 or doxorubicin alone.

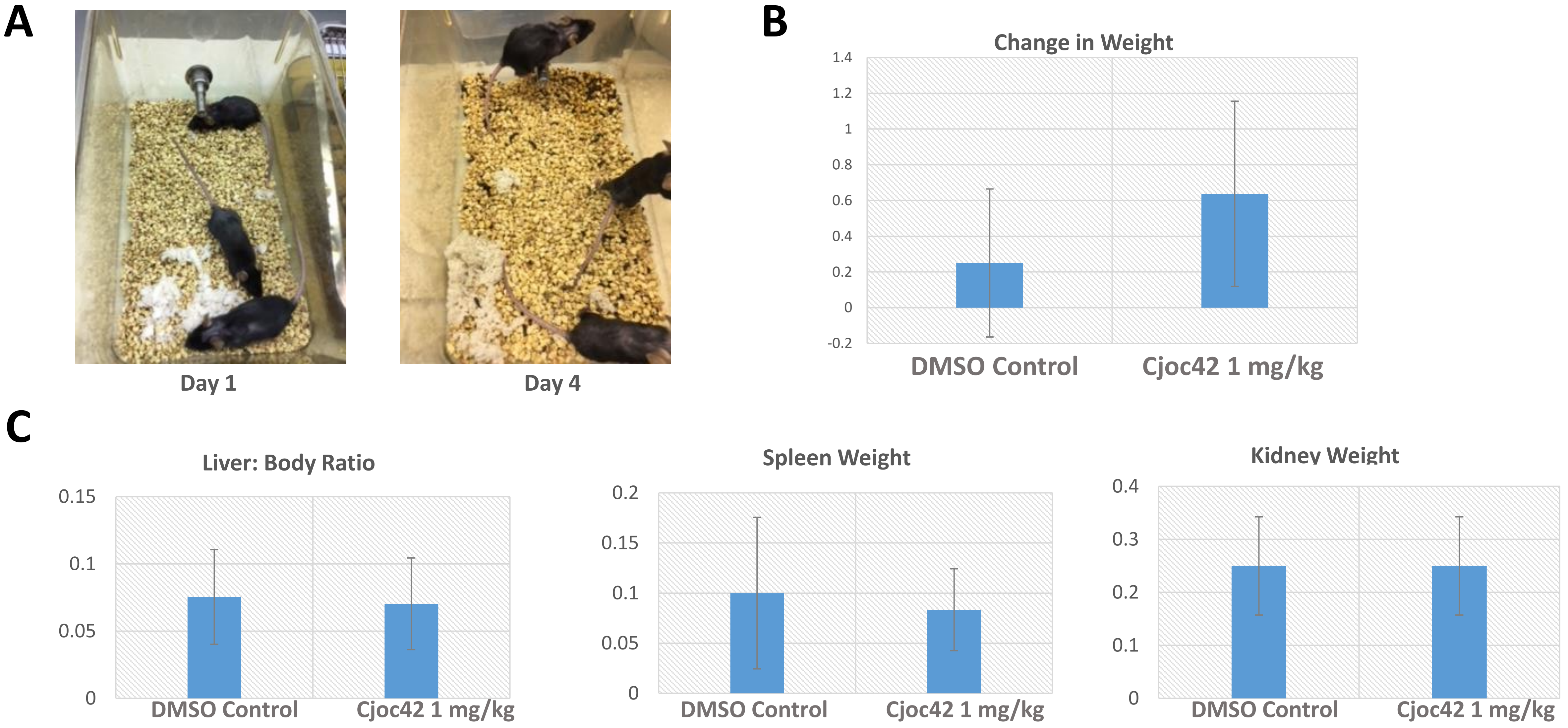

D'Souza, Supplemental Figure 6. **Additional results from the mouse toxicity study. (A)** One cage with two mice injected with cjoc42 1 mg/kg and one mouse who did not receive any injection. All three mice appear healthy throughout the course of the toxicity study including the day of injection (Day1) and the day of harvest (Day 4). **(B)** There was no difference in the change in weight between the day of injection and they day of harvest when comparing mice injected with DMSO and those injected with the highest dose of cjoc42 (1 mg/kg). Eight mice were used for each group. **(C)** There was no statistically significant difference in liver: body ratio, spleen weight, or kidney weight between DMSO control and cjoc42 1 mg/kg. Eight mice were used in each group.

Whole images of gels for Western blotting

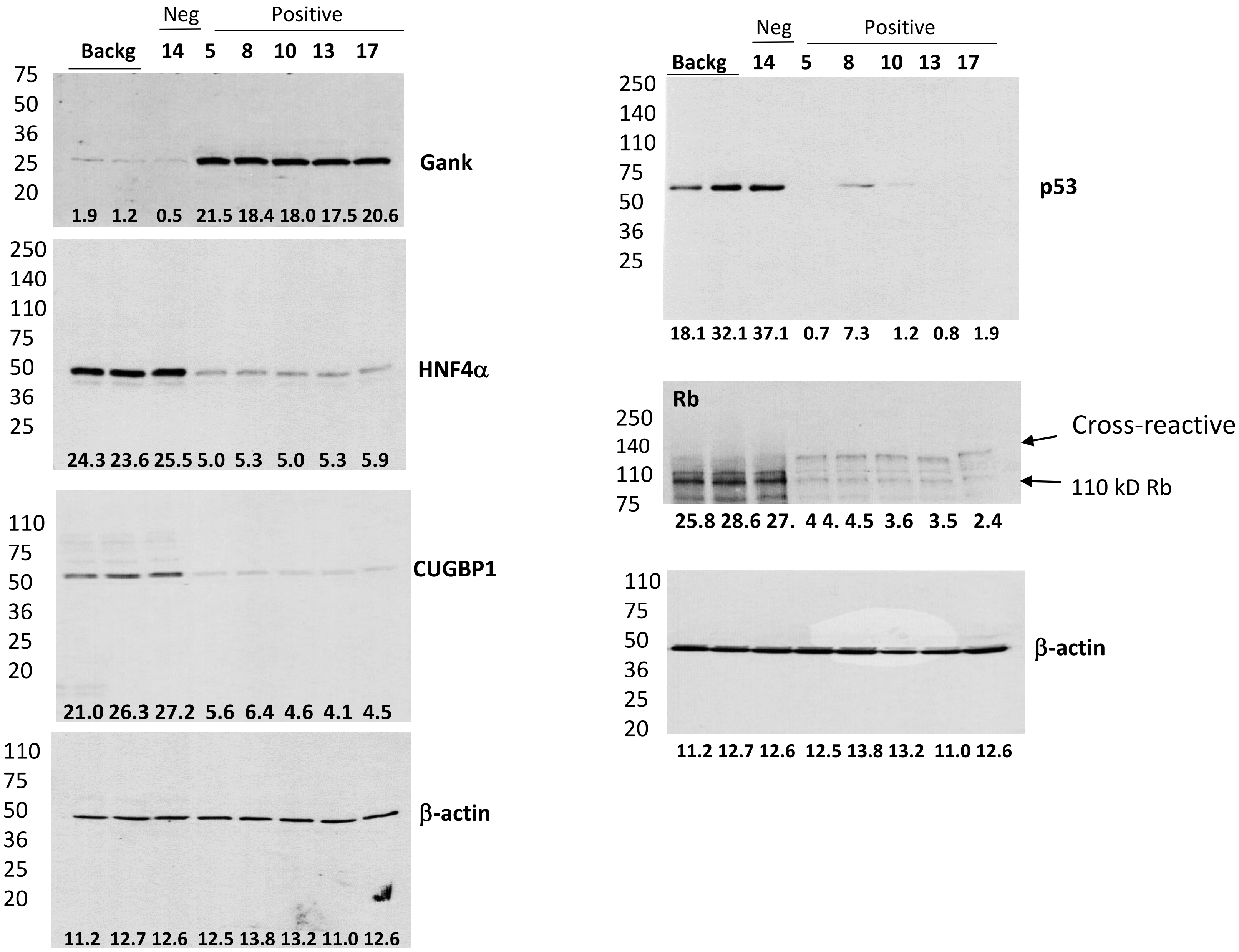

D'Souza, Supplemental Figure 7. Whole gel images of Western blots for the figure 1A.

F

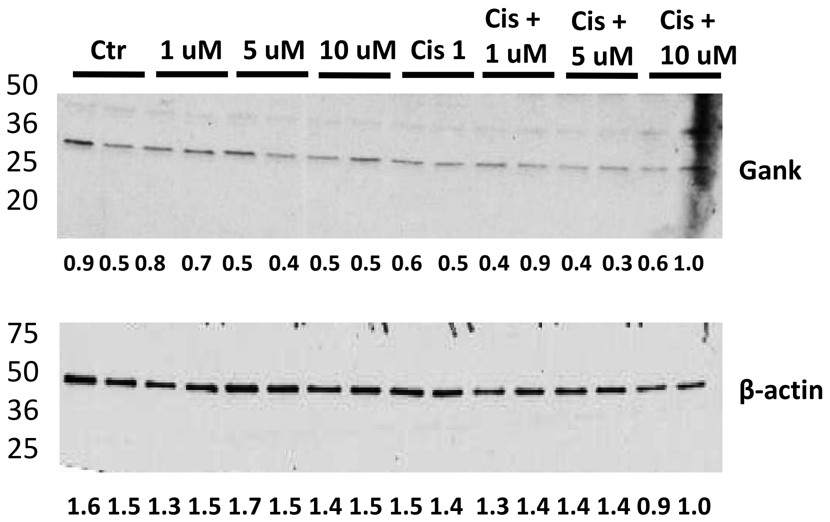

G

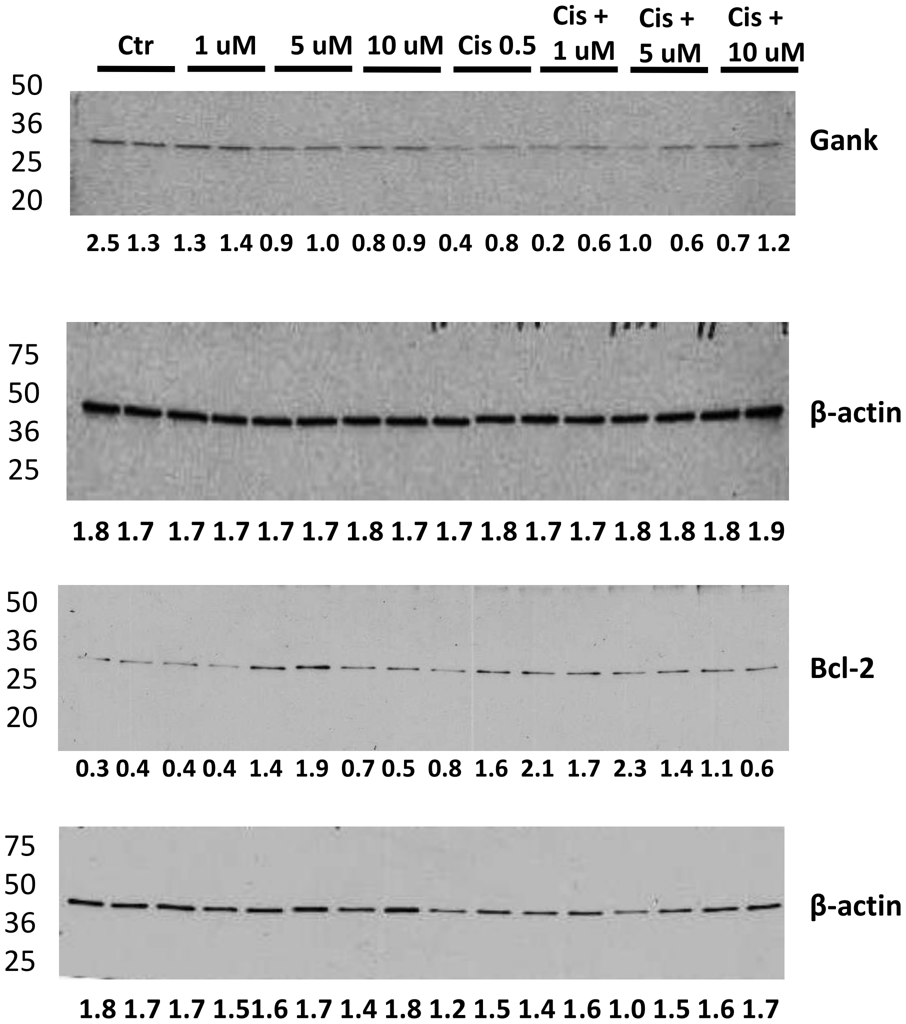

D'Souza, Supplemental Figure 8. Whole gel images of Western blots for the figure 2F and G.

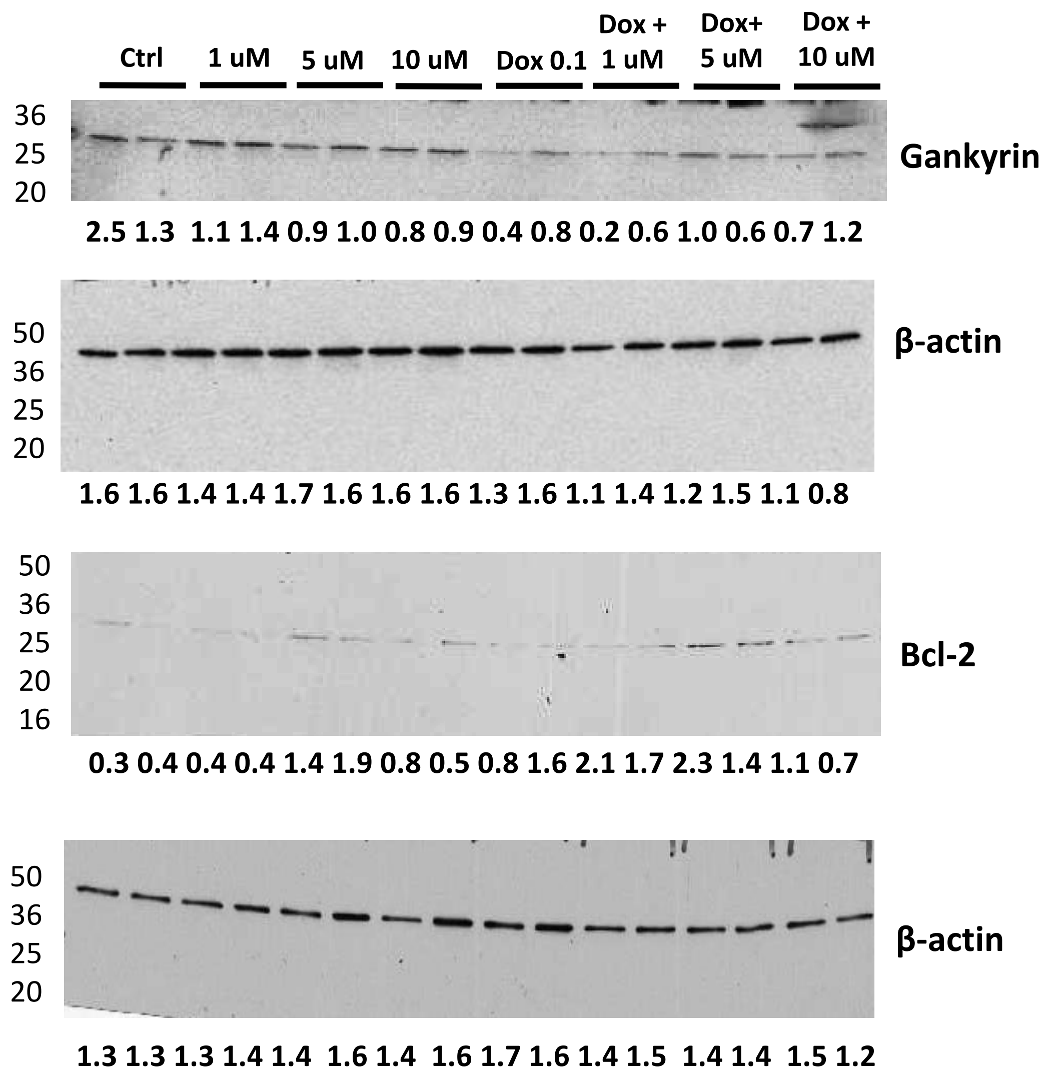

D'Souza, Supplemental Figure 9. Whole gel images of Western blots for the figure 3E.

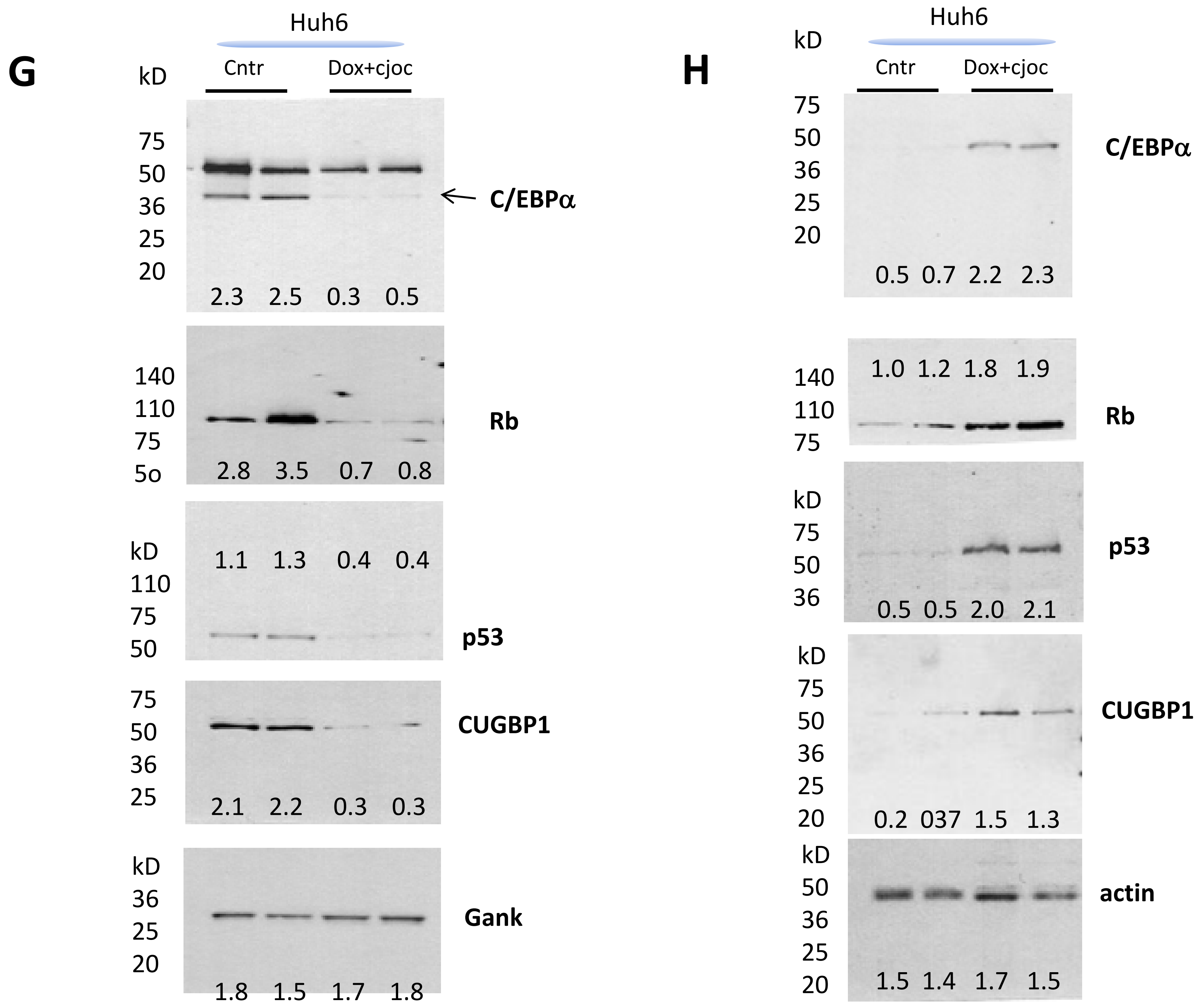

D'Souza, Supplemental Figure 10.
Whole gel images of Western blots for the figure 3G and H.

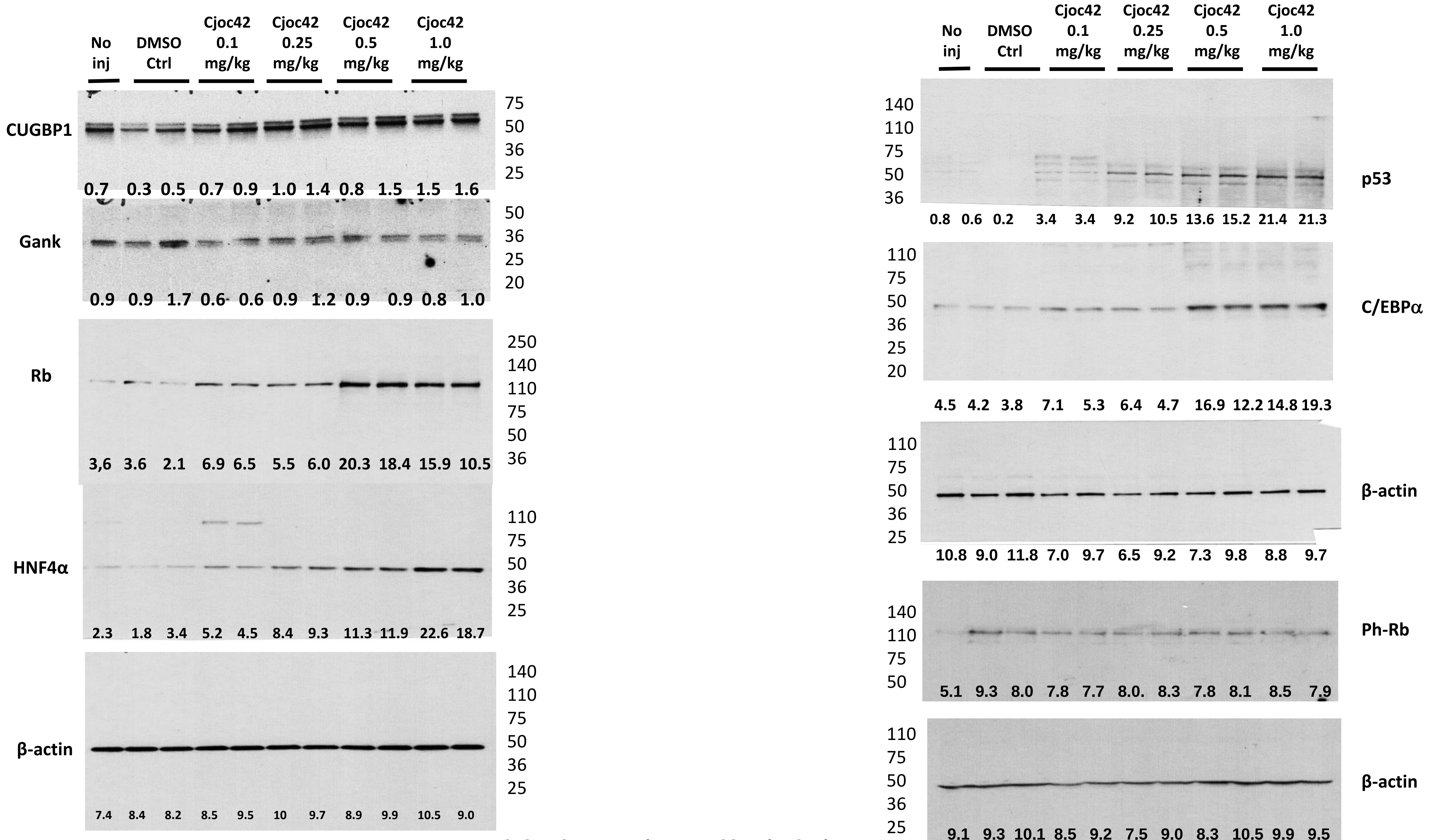

D'Souza, Supplemental Figure 11. Whole gel images of Western blots for the figure 5B.

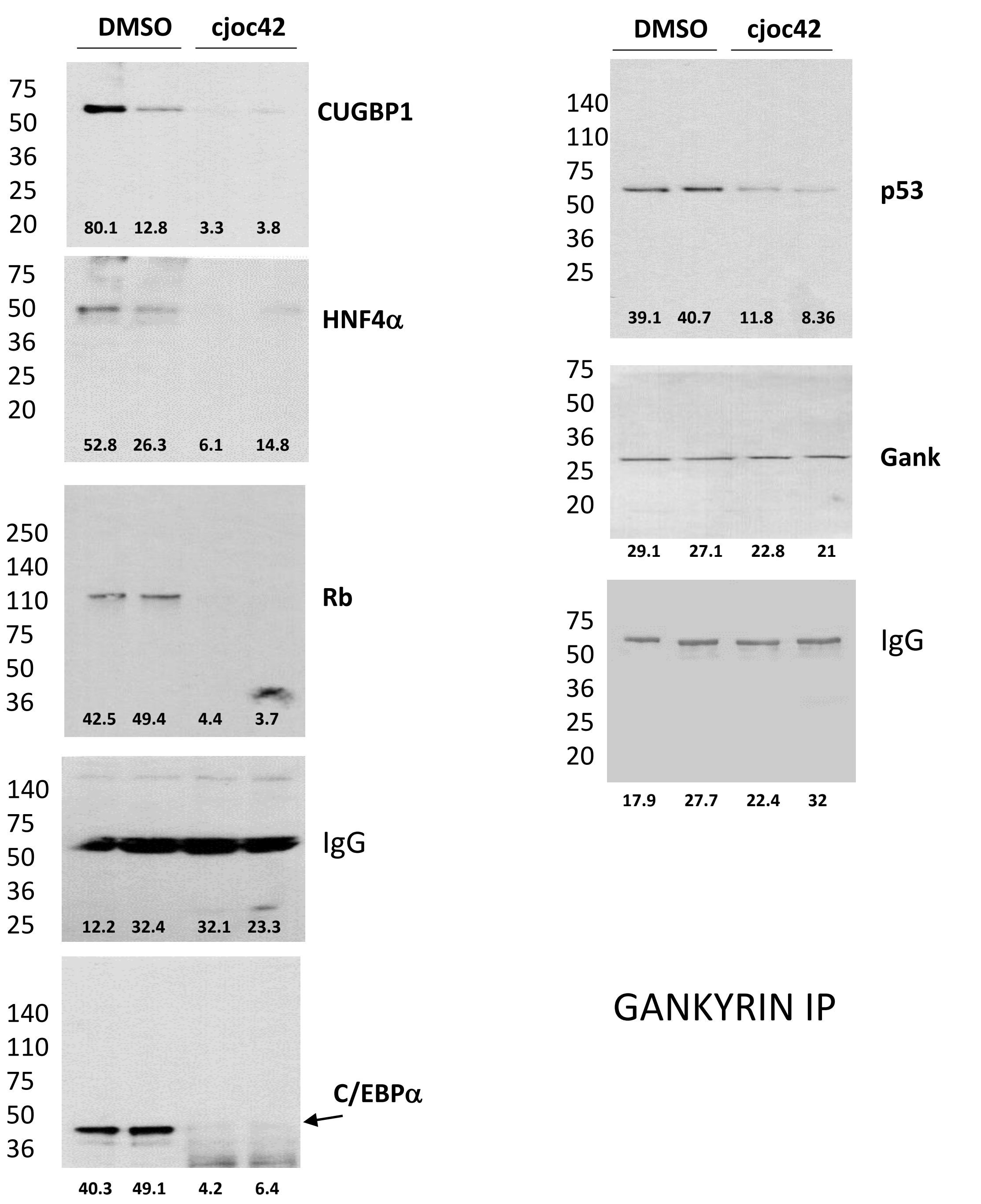

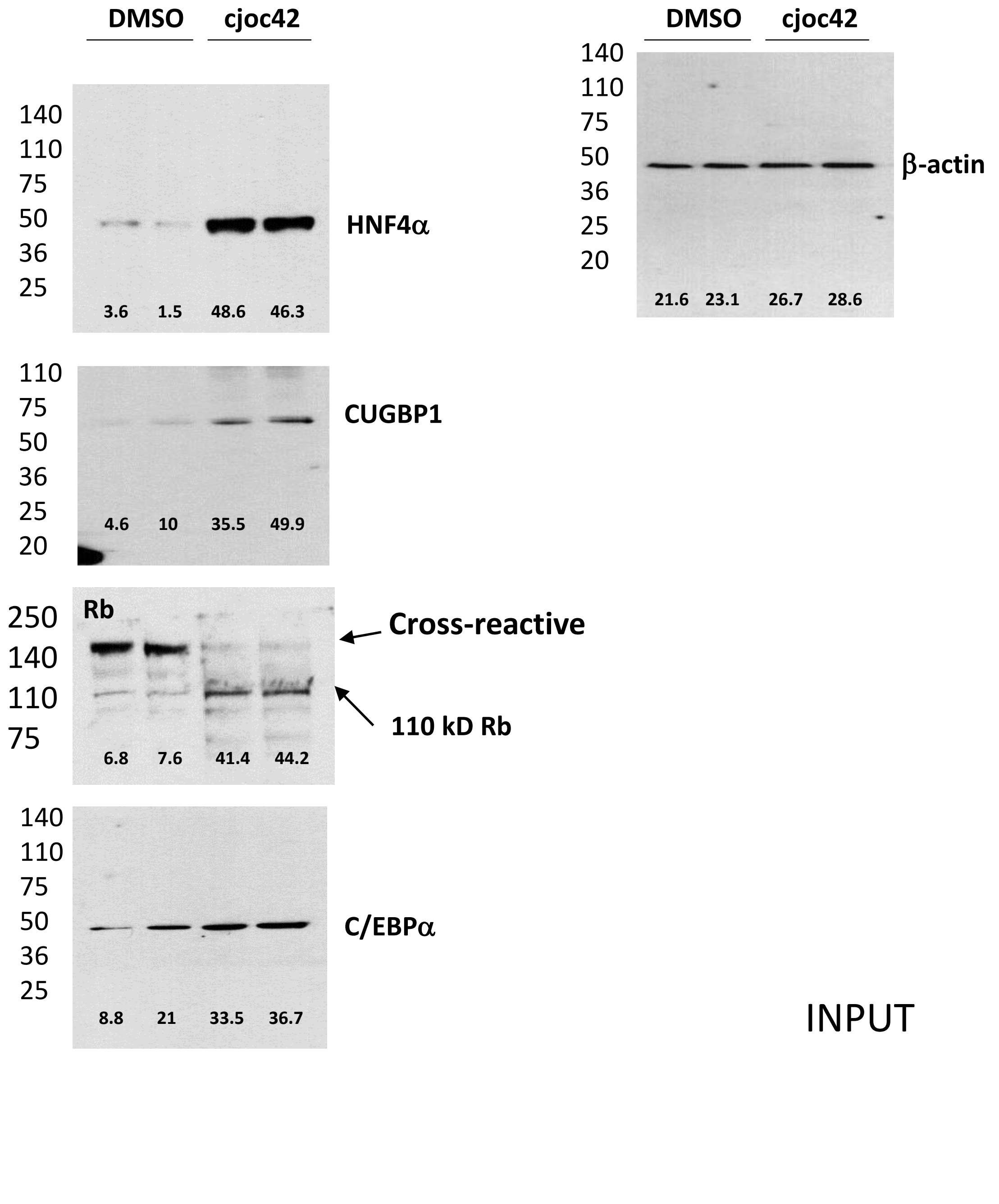

D'Souza, Supplemental Figure 12. Whole gel images of Western blots for the figure 5D.
